# GSK872 and necrostatin-1 protect retinal ganglion cells against necroptosis through inhibition of RIP1/RIP3/MLKL pathway in glutamate-induced retinal excitotoxic model of glaucoma

**DOI:** 10.1186/s12974-022-02626-4

**Published:** 2022-10-26

**Authors:** Mengyuan Liu, Haibo Li, Rongliang Yang, Dan Ji, Xiaobo Xia

**Affiliations:** 1 Eye Center of Xiangya Hospital, Central South University, Changsha, 410008 Hunan People’s Republic of China; 2 Hunan Key Laboratory of Ophthalmology, Changsha, 410008 Hunan People’s Republic of China; 3 National Clinical Research Center for Geriatric Disorders, Xiangya Hosiptal, Central South University, Changsha, Hunan People’s Republic of China

**Keywords:** GSK872, Necrostatin-1, Glaucoma, Necroptosis, NLRP3 inflammasome, Retinal ganglion cells

## Abstract

**Background:**

Glaucoma, the major cause of irreversible blindness worldwide, is characterized by progressive degeneration of retinal ganglion cells (RGCs). Current treatments for glaucoma only slow or partially prevent the disease progression, failing to prevent RGCs death and visual field defects completely. Glutamate excitotoxicity via *N*-methyl-d-aspartic acid (NMDA) receptors plays a vital role in RGCs death in glaucoma, which is often accompanied by oxidative stress and NLRP3 inflammasome activation. However, the exact mechanisms remain unclear.

**Methods:**

The glutamate-induced R28 cell excitotoxicity model and NMDA-induced mouse glaucoma model were established in this study. Cell counting kit-8, Hoechst 33342/PI dual staining and lactate dehydrogenase release assay were performed to evaluate cell viability. Annexin V-FITC/PI double staining was used to detect apoptosis and necrosis rate. Reactive oxygen species (ROS) and glutathione (GSH) were used to detect oxidative stress in R28 cells. Levels of proinflammatory cytokines were measured by qRT-PCR. Transmission electron microscopy (TEM) was used to detect necroptotic morphological changes in RGCs. Retinal RGCs numbers were detected by immunofluorescence. Hematoxylin and eosin staining was used to detect retinal morphological changes. The expression levels of RIP1, RIP3, MLKL and NLRP3 inflammasome-related proteins were measured by immunofluorescence and western blotting.

**Results:**

We found that glutamate excitotoxicity induced necroptosis in RGCs through activation of the RIP1/RIP3/MLKL pathway in vivo and in vitro. Administration of the RIP3 inhibitor GSK872 and RIP1 inhibitor necrostatin-1 (Nec-1) prevented glutamate-induced RGCs loss, retinal damage, neuroinflammation, overproduction of ROS and a decrease in GSH. Furthermore, after suppression of the RIP1/RIP3/MLKL pathway by GSK872 and Nec-1, glutamate-induced upregulation of key proteins involved in NLRP3 inflammasome activation, including NLRP3, pro-caspase-1, cleaved-caspase-1, and interleukin-1β (IL-1β), was markedly inhibited.

**Conclusions:**

Our findings suggest that the RIP1/RIP3/MLKL pathway mediates necroptosis of RGCs and regulates NLRP3 inflammasome activation induced by glutamate excitotoxicity. Moreover, GSK872 and Nec-1 can protect RGCs from necroptosis and suppress NLRP3 inflammasome activation through inhibition of RIP1/RIP3/MLKL pathway, conferring a novel neuroprotective treatment for glaucoma.

**Supplementary Information:**

The online version contains supplementary material available at 10.1186/s12974-022-02626-4.

## Introduction

Glaucoma is a major cause of irreversible blindness worldwide, affecting millions of people [1]. Glaucoma is characterized by selective loss of retinal ganglion cells (RGCs) and injury to their axons, ultimately leading to visual field defects [2]. Currently, intraocular pressure (IOP) reduction is the only proven treatment to slow or partially prevent the progression of glaucoma, but it is unable to prevent RGCs death and visual loss completely. Therefore, it is of great significance to investigate an effective way to protect RGCs from death in glaucoma.

Glutamate is the major excitatory neurotransmitter in the central nervous system (CNS) [3], mediating the transmission of visual signals between bipolar cells, photoreceptors and RGCs [4]. During the progression of glaucoma, glutamate excitotoxicity caused by inefficient removal of glutamate between synapses has been proven to play a vital role in RGCs death [5, 6]. Excessive glutamate causes overstimulation of *N*-Methyl-d-aspartate receptors (NMDARs), the primary glutamate receptors in RGCs [7, 8], leading to glutamate excitotoxicity which could induce intracellular calcium overload, mitochondrial dysfunction, oxidative stress, inflammation, and RGCs death [5, 9]. Thus, in order to demonstrate the exact mechanisms of RGCs death caused by glutamate excitotoxicity, we established a mouse model of glaucoma by intravitreal injection of *N*-Methyl-d-aspartate (NMDA), the agonist molecule of glutamate that selectively binds to NMDARs. Moreover, we used R28 cells, a retinal precursor cell line with biological properties similar to RGCs, which is commonly applied for in vitro studies of the neuroprotection and physiological function of RGCs [10–12], to establish a glutamate excitotoxicity model in vitro.

Multiple types of cell death are involved in RGCs loss in the progression of glaucoma, of which apoptosis and necrosis are the most widely researched [13–15], whereas necroptosis has rarely been given attention to in previous studies. Recently, an increasing number of reports have posited that necroptosis is involved in neuronal death in several neurodegenerative disorders, and inhibition of necroptosis can offer a neuroprotective effect [16, 17]. Necroptosis is a novel form of programmed cell death that can be activated by various stimuli, such as ischemia/reperfusion injury, oxidative stress damage, and inflammation [18, 19]. Morphologically, necroptosis is accompanied by cell swelling, vacuolization of cytoplasmic organelles, fragmentation of chromatin, rupture of plasma membrane and cell lysis, which is similar to necrosis [20, 21]. Necroptosis is mainly regulated by the receptor interacting protein kinase 1 (RIP1)/receptor interacting protein kinase 3 (RIP3)/mixed lineage kinase-like domain protein (MLKL) pathway [22]. Upon stimulation, RIP1 initiates necroptosis by activating and interacting with RIP3 to form necrosome [20]. Then, activated RIP3 phosphorylates MLKL, the terminal executor of necroptosis, which ultimately leads to cell lysis [23–25]. However, necroptosis can be attenuated by specific inhibitors, such as the RIP1 inhibitor necrostatin-1 (Nec-1) and the RIP3 inhibitor GSK872. GSK872 and Nec-1 have been reported to show neuroprotective effects in some neurodegenerative diseases, such as Alzheimer’s disease, by promoting neuronal survival and inhibiting neuroinflammation [26–29]. However, the protective effects of GSK872 and Nec-1 on RGCs protection in glaucoma remain unknown.

Nucleotide-binding leucine-rich repeat-containing receptor family, pyrin domain containing 3 (NLRP3) inflammasome, an innate immune signaling receptor regulating inflammatory responses, is closely associated with neuroinflammation in glaucoma [30, 31]. Upon activation by stimuli such as reactive oxygen species (ROS), it leads to the cleavage of caspase-1 and subsequent secretion of the mature forms of the proinflammatory cytokines interleukin-1β (IL-1β) and interleukin-18 (IL-18), which can promote neuronal death in glaucoma, along with other neurodegenerative diseases [32–36]. Thus, inhibition of NLRP3 inflammasome may be a potential strategy to prevent RGCs death in glaucoma. Some studies have focused on the associations between NLPR3 inflammasome and glaucoma, it is reported that NLRP3 inflammasome-induced release of IL-1β plays an important role in neuroinflammation and RGCs death in glaucoma and many cells and pathways participate in this process, such as microglia and NF-κβ pathway [37]. Besides, the study has showed that NLRP3 inflammasome activation is involved in RGCs death by inducing pyroptosis in the retina exposed to ocular hypertension injury [38]. Nevertheless, the exact mechanisms of NLRP3 inflammasome activation in the progression of glaucoma remain unknown and need to be further studied. Recent studies have provided some evidence that necroptosis-related proteins such as RIP3 and MLKL may be involved in regulating NLRP3 inflammasome activation [39–41]. Activated MLKL can translocate to the cell membranes and trigger iron release, which may contribute to NLRP3 inflammasome activation [42–44]. Additionally, the inflammation induced by NLRP3 inflammasome activation could promote necroptosis as well. Therefore, it is necessary to investigate the interactions occurring between necroptosis and NLRP3 inflammasome activation in glaucoma, which may provide a promising target for RGCs protection and neuroinflammatory attenuation.

The present study aimed to investigate the potential role of necroptosis in glutamate-induced RGCs death and the underlying mechanisms, as well as the neuroprotective effects of Nec-1 and GSK872 on glutamate excitotoxicity in RGCs. In this study, we found that necroptosis played a crucial role in RGCs death in the glutamate-induced excitotoxic model of glaucoma in vitro and in vivo. Moreover, GSK872 and Nec-1 significantly prevented RGCs from death, inhibited oxidative stress and NLRP3 inflammasome activation through suppression of RIP1/RIP3/MLKL pathway. To the best of our knowledge, this is the first study to elucidate the association between necroptosis and NLRP3 inflammasome in glaucoma. Our investigation sheds new light on the mechanism of RGCs death caused by glutamate excitotoxicity, highlighting the potential neuroprotective value of GSK872 and Nec-1 in glaucoma treatment.

## Results

### Necroptosis played a vital role in RGCs death induced by glutamate excitotoxicity

**Fig. 1:**
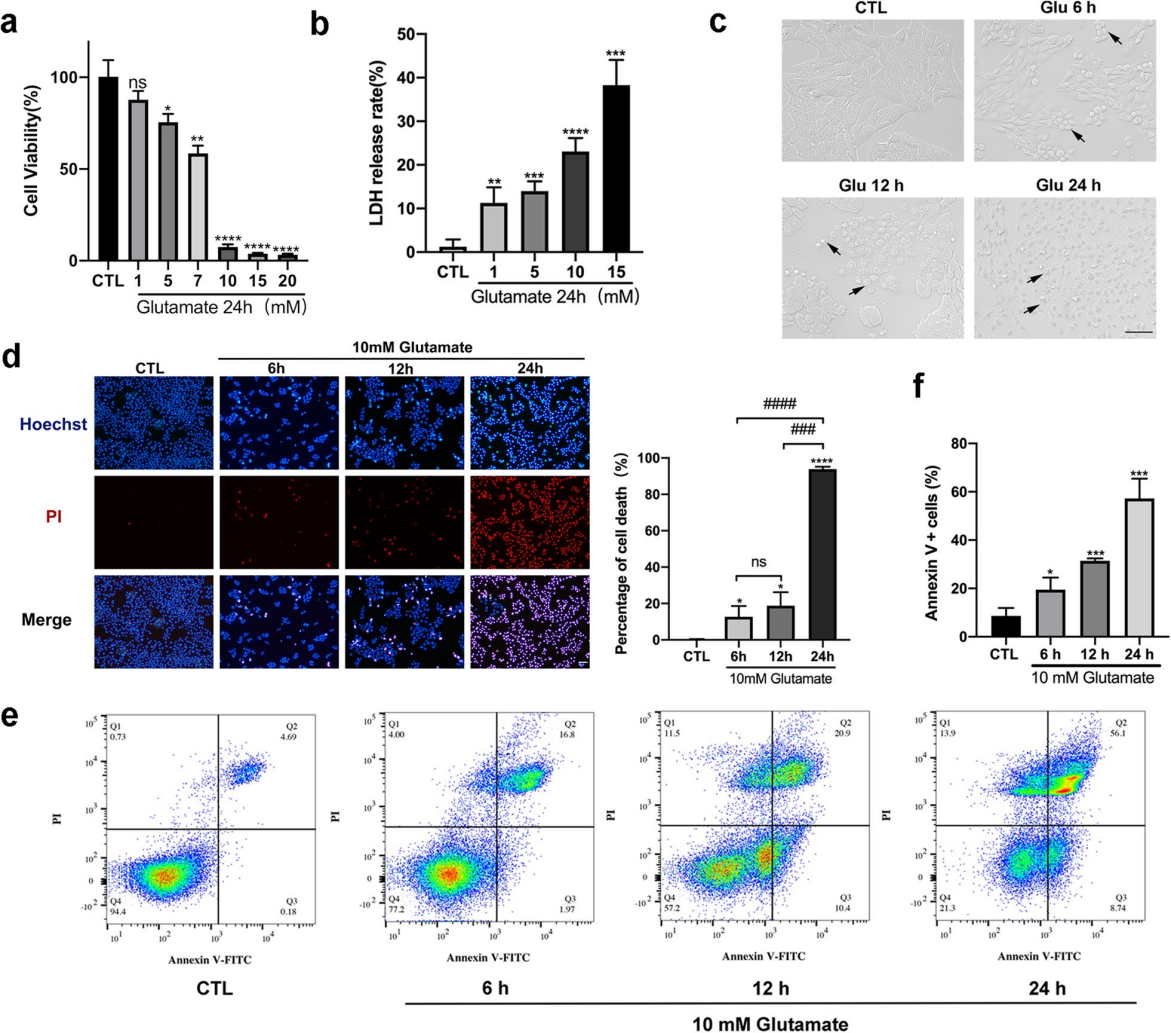
Necroptosis induced by glutamate in R28 cells. **a** CCK-8 assay for R28 cells treated with different concentrations of glutamate for 24 h. **b** LDH release assay was used to test the cell death rate of R28 cells treated with different concentrations of glutamate for 24 h. **c** Morphology of R28 cells exposed to 10 mM glutamate for 6 h, 12 h, and 24 h. The cells were photographed under a light microscope. The black arrows indicate the morphological changes of R28 cells induced by glutamate. Scale bar = 50 μm. **d** Hoechst–PI dual staining assay was used to test the cell death rate of R28 cells after administration of 10 mM glutamate for 6 h, 12 h, and 24 h. Cell death rate increased in a time-dependent manner. Scale bar = 50 μm. **e**, **f** Flow cytometry with Annexin V/PI double staining was used to detect the percentage of apoptosis and necrosis in R28 cells after glutamate treatment. CTL: the control group. The results were recorded as mean ± SD from at least three independent experiments. *p < 0.05, **p < 0.01, ***p < 0.001, ****p < 0.0001 versus control group; ^###^*p* < 0.001, ^####^*p* < 0.0001. ns: not significant

To clarify whether glutamate could suppress cell survival, we used a CCK-8 kit to test the cell viability of R28 cells treated with a series of concentrations of glutamate for 24 h (Fig. 1a). The results revealed that glutamate inhibited R28 cell survival in a dose-dependent manner; when treated with 10 mM glutamate, cell viability was markedly reduced compared with the control group. To further verify the toxicity of glutamate on R28 cells, we used a lactate dehydrogenase (LDH) release assay to detect the cell death rate (Fig. 1b). The results showed that the release of LDH was increased noticeably as the glutamate concentration increased, and 10 mM glutamate significantly inhibited cell survival, which was consistent with the results of the CCK-8 test. Therefore, we chose 10 mM as the concentration for the subsequent in vitro studies.

**Fig. 2:**
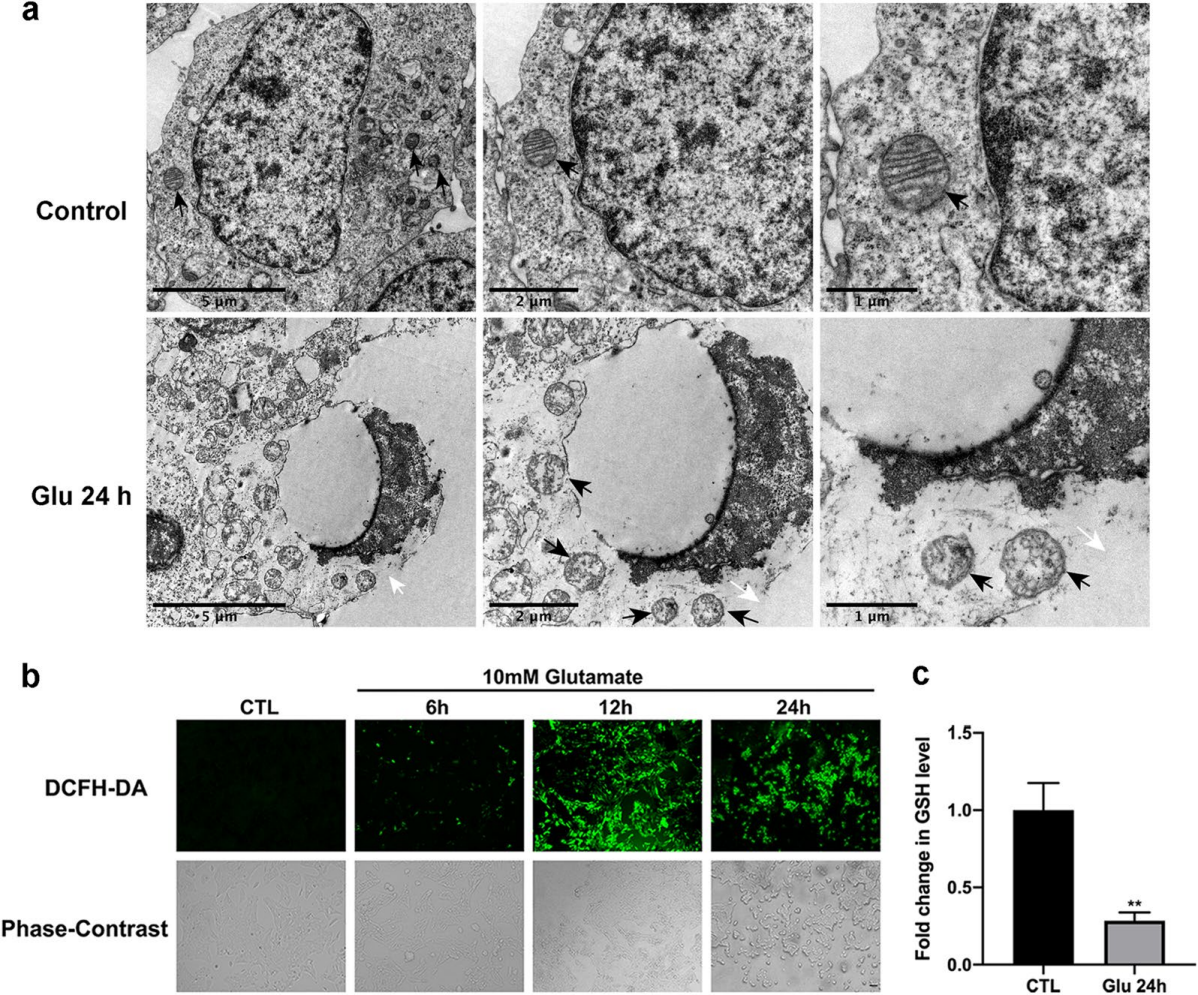
Necroptotic morphology and overproduction of ROS in R28 cells induced by glutamate. **a** The typical morphological changes of necroptosis were detected in glutamate-treated R28 cells by transmission electron microscopy (TEM), including cell swelling, vesiculation of cytoplasmic organelles and rupture of the plasma membrane. Black arrows indicate mitochondria; white arrows indicate rupture of plasma membrane. **b** Representative phase-contrast and fluorescence microscope images of R28 cells incubated with the ROS probe DCFH-DA after exposure to glutamate for different durations. The green fluorescence intensity indicated the level of intracellular ROS. Scale bar = 50 μm. **c** Cellular GSH levels were measured at 24 h after glutamate treatment. CTL: the control group; Glu: glutamate. The results were recorded as mean ± SD from at least three independent experiments. **p < 0.01 versus control group

Next, we used a light microscope to observe the morphological changes in R28 cells treated with 10 mM glutamate at several time points (Fig. 1c). Normal R28 cells were spindle-shaped with tight junctions between cells. After exposure to glutamate for 6 h, some cells began to swell. At 12 h, the majority of the cells exhibited morphological changes, and the connections between cells gradually disappeared. After 24 h of treatment, the R28 cells had lost their normal morphology and began to rupture, showing typical necrotic morphological changes. Furthermore, Hoechst 33342/propidium iodide (PI) double staining assay was used to detect cell mortality at different time points of glutamate treatment (Fig. 1d). We found that the cell death rate was significantly increased after 24 h of glutamate treatment. Moreover, flow cytometry with Annexin V-FITC/PI double staining revealed that R28 cells treated with glutamate underwent apoptosis and necrosis. From 6 to 24 h, the percentage of Annexin V^+^/PI^−^ (early apoptosis) cells and Annexin V^+^/PI^+^ (necrosis and late apoptosis) cells were significantly increased in a time-dependent manner (Fig. 1e, f). Thus, a 24-h treatment duration was adopted in further studies. Transmission electron microscopy (TEM) detected that R28 cells incubated with glutamate for 24 h exhibited a typical necroptotic morphology characterized by swollen cells, irregular nuclei, dissolved chromatin, vacuolization of cytoplasmic organelles, membrane rupture and release of cellular contents (Fig. 2a).

**Fig. 3:**
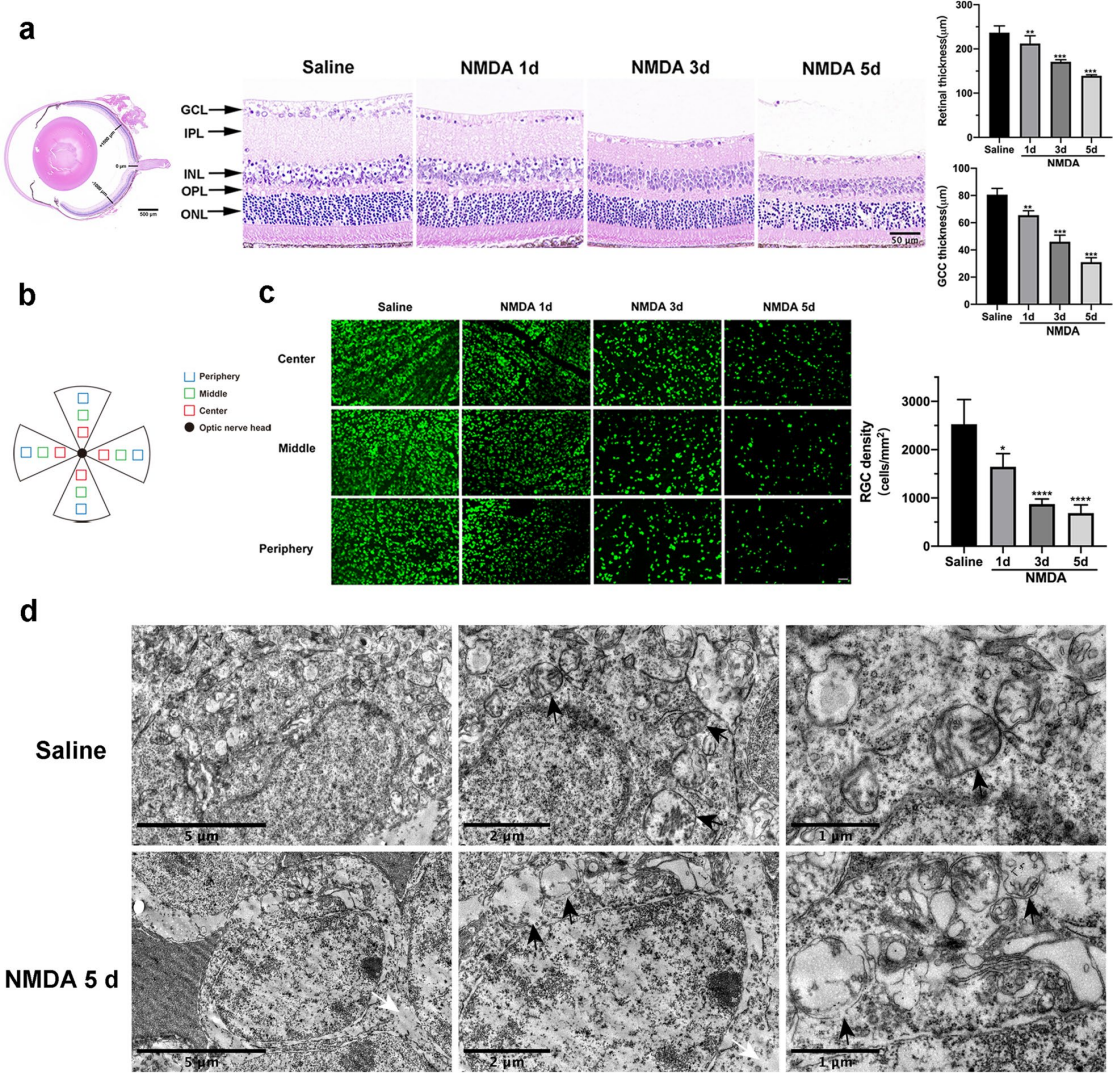
NMDA-induced retinal damage and necroptosis of RGCs in mice. **a** HE staining and quantitative analysis of total retinal thickness and GCC thickness in retina tissue harvested at different times post intravitreal injection of NMDA or saline. The thickness was measured in the area 1 mm around the optic nerve. Scale bar = 50 μm. **b** Schematic illustration of the retinal area. RGCs were quantified in three areas (center, middle, periphery) in each of the four quadrants of the retina. **c** Immunofluorescence staining of flat-mounted mice retinas with RBPMS (green) and quantification of RBPMS-positive RGCs at different times after NMDA injury. Scale bar = 50 μm. **d** Transmission electron microscopy (TEM) detected typical necroptotic morphology in RGCs after NMDA injury, including cell swelling, vesiculation of cytoplasmic organelles and rupture of the plasma membrane. Black arrows indicate mitochondria; white arrows indicate rupture of plasma membrane. GCL: ganglion cell layer; IPL: inner plexiform layer; INL: inner nuclear layer; OPL: outer plexiform layer; ONL: outer nuclear layer; GCC: ganglion cell complex. The results were recorded as mean ± SD from at least three independent experiments. *p < 0.05, **p < 0.01, ***p < 0.001, ****p < 0.0001 versus saline group

To investigate glutamate-induced excitotoxicity in vivo, a glaucoma mouse model was established by intravitreal injection of NMDA. Hematoxylin and eosin (HE) staining was used to detect the retinal morphological changes caused by NMDA. The total retinal thickness and ganglion cell complex (GCC) thickness were significantly decreased at different time points after intravitreal injection of NMDA compared with the saline-treated group (Fig. 3a). Moreover, we used whole-mount staining of the retina using RNA-binding protein with multiple splicing (RBPMS) to examine the number of RGCs at 1, 3, and 5 days after NMDA treatment. The density of RGCs was markedly reduced in a time-dependent manner post-NMDA injection (Fig. 3b, c). Thus, we chose 5 days for all subsequent experiments in vivo. To demonstrate the role of necroptosis in RGCs death, we used TEM to observe the morphological changes in RGCs after NMDA injection. As shown in Fig. 3d, RGCs in the NMDA group exhibited apparent necroptotic changes, including enlarged mitochondria, vacuolization of cytoplasmic organelles, followed by extensive rupture of plasma and nuclear membrane and leakage of cell contents. Taken together, these results indicate that necroptosis plays an important role in retinal injury and RGCs death caused by glutamate excitotoxicity.

### Glutamate-induced overproduction of ROS and upregulation of proinflammatory cytokines in R28 cells

Given that glutamate-induced cell death is often accompanied by oxidative stress and inflammation [4, 45], we measured the levels of ROS and proinflammatory cytokines in R28 cells subjected to 10 mM glutamate at different time points to verify whether they contributed to the process of necroptosis. Fluorescence microscopy revealed that the intracellular ROS level was significantly increased in comparison with the control group in R28 cells from 6 to 24 h of glutamate incubation, peaking at 12 h (Fig. 2b). To further investigate the extent of oxidative stress, cellular glutathione (GSH) levels, as another marker of the oxidation–reduction system, were measured at 24 h after glutamate administration. The results showed that cellular GSH was significantly decreased after incubation with glutamate compared with the control group (Fig. 2c). Moreover, we performed qRT-PCR to test the changes in mRNA levels of proinflammatory factors caused by glutamate in R28 cells. As shown in Additional file 1: Fig. S1, the expression levels of TNF-α, IL-6, and IL-1β were obviously upregulated after treatment with glutamate. Collectively, the results demonstrate that oxidative stress and inflammation are involved in glutamate-induced excitotoxic injury in R28 cells.

### RIP1/RIP3/MLKL pathway and NLRP3 inflammasome were activated in glutamate-induced retinal excitotoxic model

**Fig. 4:**
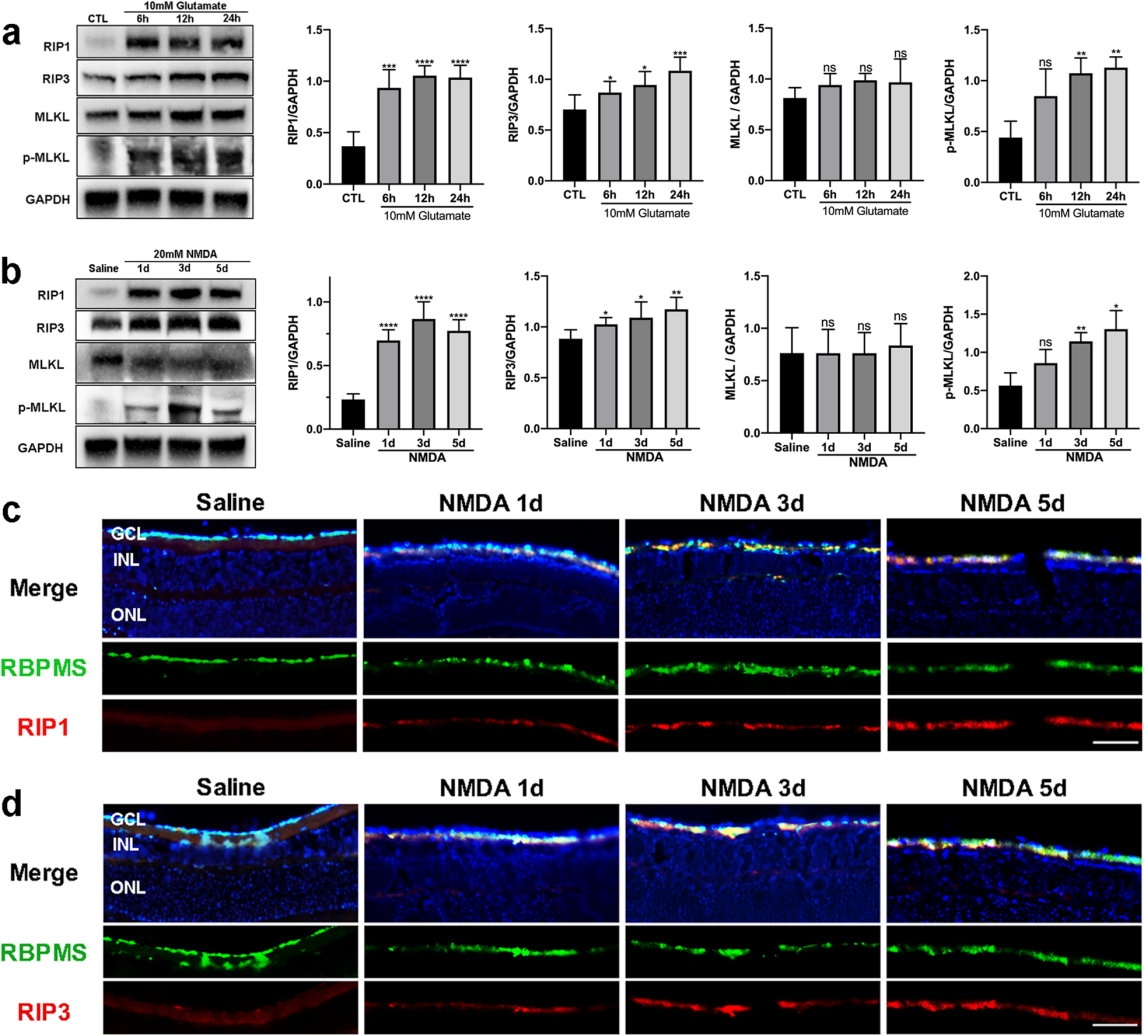
Activation of the RIP1/RIP3/MLKL pathway in glutamate-induced excitotoxic model. **a** Protein levels of RIP1, RIP3, MLKL and p-MLKL (phospho S345) in R28 cells after treatment with glutamate for 6 h, 12 h, and 24 h. **b** Protein levels of RIP1, RIP3, MLKL and p-MLKL (phospho S345) in the mouse retina after intravitreal administration of NMDA for 1, 3, and 5 days. **c** Representative immunofluorescence microphotographs of retinal sections stained with RBPMS (green), RIP1 (red), and DAPI (blue) post-NMDA injection. Scale bar = 50 μm. (d) Representative immunofluorescence microphotographs of retinal sections stained with RBPMS (green), RIP3 (red), and DAPI (blue) post-NMDA injection. Scale bar = 50 μm. GCL: ganglion cell layer; INL: inner nuclear layer; ONL: outer nuclear layer; CTL: the control group. The results were recorded as mean ± SD from at least three independent experiments. *p < 0.05, **p < 0.01, ***p < 0.001, ****p < 0.0001 versus control or saline group. ns: not significant

Considering that RIP1, RIP3, and MLKL are the major signals of necroptosis, we applied western blot analysis to test the protein levels of the three necroptosis-associated molecules in R28 cells treated with glutamate (Fig. 4a). At 6 h, 12 h and 24 h of glutamate treatment, the expression level of RIP1 was significantly upregulated compared with the control group. Moreover, there was a time-dependent increase in the protein level of RIP3 as well. As the executor of necroptosis, the expression level of p-MLKL was also markedly increased as the treatment was prolonged and peaked at 24 h, while there were not significant changes in MLKL expression. In summary, the protein levels of RIP1, RIP3 and p-MLKL were positively correlated with the degree of necroptosis mentioned previously, indicating that glutamate could mediate the activation of the RIP1/RIP3/MLKL pathway in R28 cells, ultimately resulting in necroptosis. Similarly, in the mouse model of glaucoma, the expression levels of RIP1 and RIP3 were significantly increased at 1, 3, and 5 days after NMDA injection compared with the saline-treated group (Fig. 4b). Meanwhile, the level of p-MLKL was markedly increased at 5 days post-NMDA injury. Furthermore, immunostaining of retinal sections was applied to detect the expressions of RIP1 and RIP3 in the GCL, and RBPMS was used as a marker of RGCs. This showed that NMDA treatment induced significant upregulation of RIP1 and RIP3 in RGCs at different time points (Fig. 4c, d). Collectively, the results indicate that RIP1/RIP3/MLKL activation was involved in RGCs necroptosis caused by glutamate excitotoxicity.

**Fig. 5:**
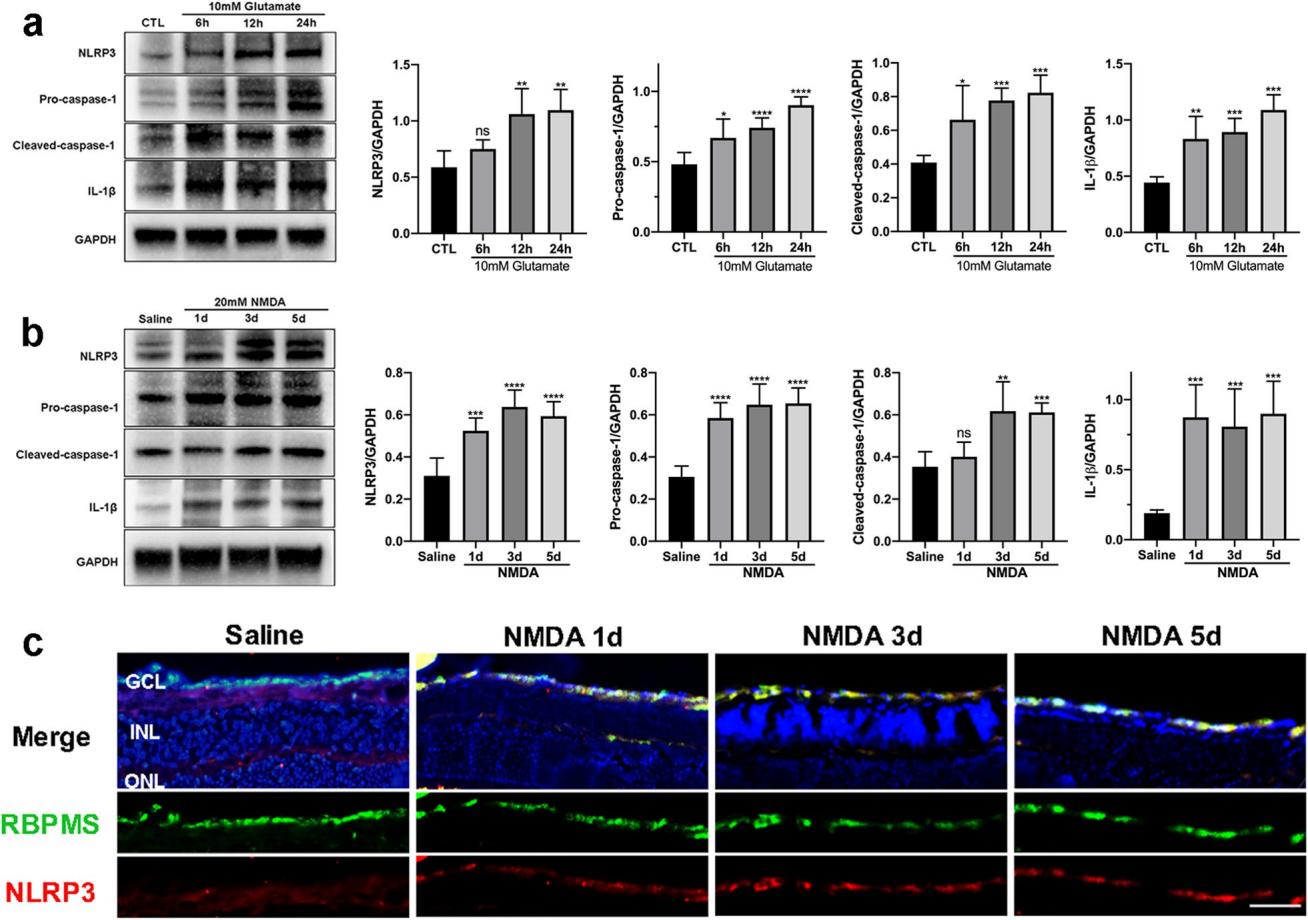
Activation of NLRP3 inflammasome triggered by glutamate excitotoxicity. **a** Protein levels of NLRP3 and NLRP3-related proteins in R28 cells after glutamate treatment. **b** Protein levels of NLRP3 and NLRP3-related proteins in the mouse retina after NMDA injury. **c** Representative immunofluorescence microphotographs of retinal sections stained with RBPMS (green), NLRP3 (red), and DAPI (blue) post-NMDA injection. Scale bar = 50 μm. GCL: ganglion cell layer; INL: inner nuclear layer; ONL: outer nuclear layer; CTL: the control group. The results were recorded as mean ± SD from at least three independent experiments. *p < 0.05, **p < 0.01, ***p < 0.001, ****p < 0.0001 versus control or saline group. ns: not significant

Studies have demonstrated that necroptotic pathway activation is often accompanied by NLRP3 inflammasome activation [41, 46]. Therefore, we detected expression changes of NLRP3 and its downstream proteins after glutamate treatment for different durations to investigate whether NLRP3 inflammasome activation was involved in glutamate-induced retinal injury. As shown in Fig. 5a, in response to glutamate stimulation, the expression of NLRP3 was markedly elevated in R28 cells at 12 h and 24 h. Moreover, the protein levels of pro-caspase-1, cleaved-caspase-1, and IL-1β were also significantly increased after glutamate treatment. Consistent with the in vitro results, retinal expression levels of NLRP3 and its downstream proteins were markedly upregulated after intravitreal NMDA administration in vivo (Fig. 5b). Meanwhile, immunofluorescence analysis showed increased expression levels of NLRP3 in RGCs of NMDA-damaged retinas compared with the saline-injected group (Fig. 5c). Since cleaved-caspase-1 and IL-1β are biomarkers of NLRP3 inflammasome activation, these observations suggest that glutamate excitotoxicity induces activation of NLRP3 inflammasome while it triggers necroptosis of RGCs in vitro and in vivo.

### GSK872 and Nec-1 attenuated glutamate-induced RGCs death and retinal damage by inhibiting the RIP1/RIP3/MLKL pathway

**Fig. 6:**
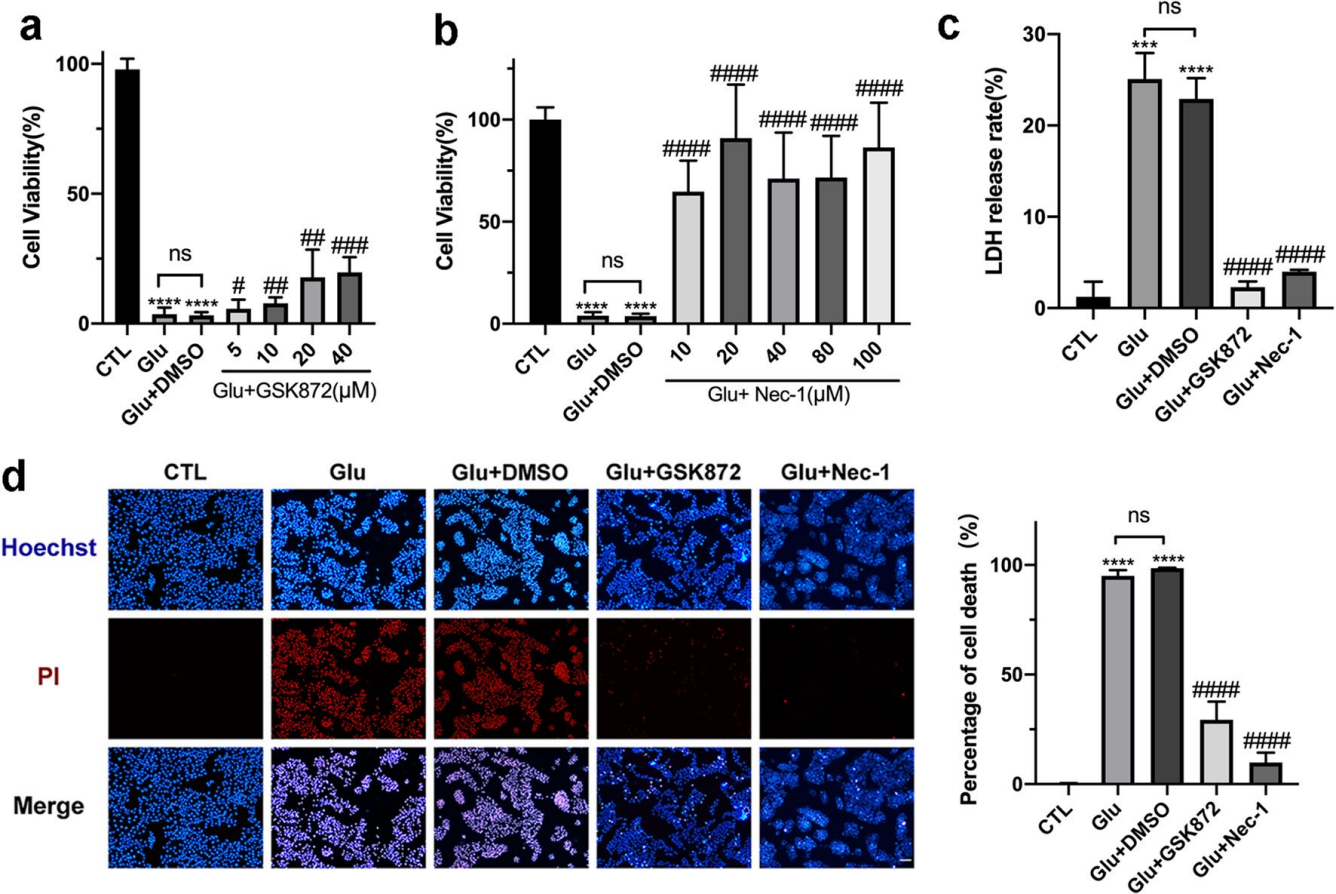
GSK872 and Nec-1 protected R28 cells from death triggered by glutamate. **a** CCK-8 assay of R28 cells treated with 10 mM glutamate and different concentrations of GSK872 for 24 h. **b** CCK-8 assay of R28 cells treated with 10 mM glutamate and different concentrations of Nec-1 for 24 h. **c** GSK872 and Nec-1 apparently blocked LDH release induced by glutamate in R28 cells. **d** Hoechst–PI dual staining assay and statistical analysis of PI-positive cells after 24 h treatment with 10 mM glutamate and 40 μM GSK872 or 20 μM Nec-1. Scale bar = 50 μm. CTL: the control group; Glu: glutamate. The results were recorded as mean ± SD from at least three independent experiments. ***p < 0.001, ****p < 0.0001 versus control; ^#^*p* < 0.05, ^##^*p* < 0.01, ^###^*p* < 0.001, ^####^*p* < 0.0001 versus Glu + DMSO group. ns: not significant

**Fig. 7:**
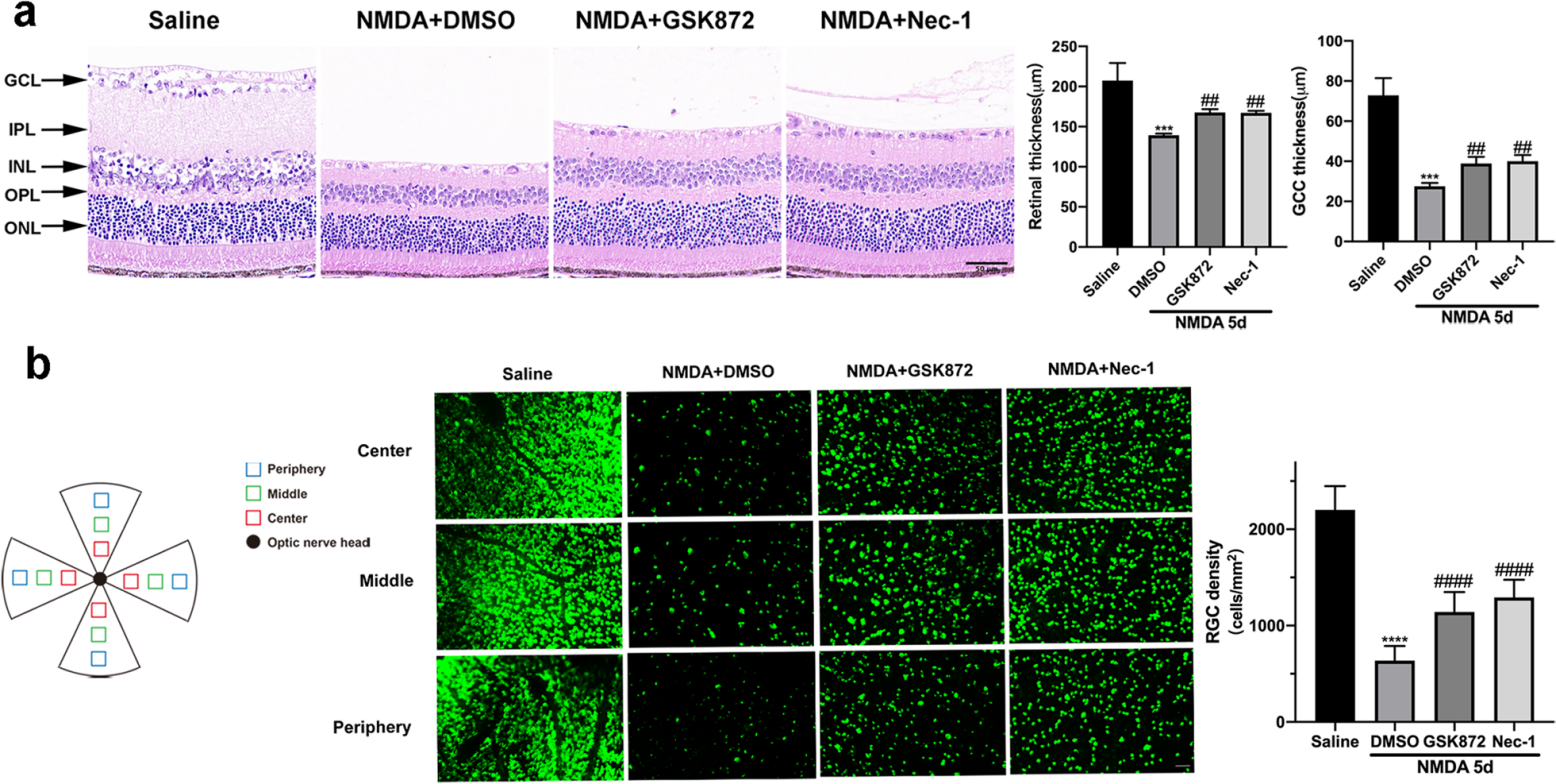
GSK872 and Nec-1 inhibited RGCs necroptosis and retinal injury in the glutamate-induced excitotoxic mouse model. **a** HE staining of retinal sections and quantitative measurement of total retinal thickness and GCC thickness after intravitreal administration of NMDA with GSK872 or Nec-1 for 5 days. Scale bar = 50 μm. **b** Immunofluorescence staining of retinal whole-mounts with RBPMS (green) and quantification of RBPMS-positive RGCs at 5 days post intravitreal injection of NMDA with GSK872 or Nec-1. RGCs were quantified in three areas (center, middle, periphery) in each of the four quadrants of the retina. Scale bar = 50 μm. GCL: ganglion cell layer; IPL: inner plexiform layer; INL: inner nuclear layer; OPL: outer plexiform layer; ONL: outer nuclear layer; GCC: ganglion cell complex. The results were recorded as mean ± SD from at least three independent experiments. ***p < 0.001, ****p < 0.0001 versus saline group; ^##^*p* < 0.01, ^####^*p* < 0.0001 versus NMDA + DMSO group. ns: not significant

To further verify the key role of the RIP1/RIP3/MLKL pathway in glutamate-induced necroptosis of RGGs, we suppressed RIP1 and RIP3 with their specific inhibitors, Nec-1 and GSK-872, respectively. A CCK-8 assay was applied to the R28 cells after treatment with glutamate and different concentrations of GSK872 or Nec-1 for 24 h (Fig. 6a, b). The results showed that GSK872 promoted cell viability in a dose-dependent manner and peaked at 40 μM. Meanwhile, Nec-1 had a more significant effect on promoting cell survival than GSK872; a 20 μM treatment of Nec-1 markedly improved cell viability. Thus, 40 μM GSK872 and 20 μM Nec-1 were selected for the subsequent in vitro studies. As shown in Fig. 6c, the LDH release was also obviously attenuated in the cells treated with GSK872 or Nec-1. Moreover, the Hoechst/PI staining showed that GSK872 and Nec-1 significantly decreased R28 cell death caused by glutamate (Fig. 6d). Furthermore, we investigated the neuroprotective effects of GSK872 and Nec-1 in vivo. As shown in Fig. 7a, intravitreal injection of 80 μM GSK872 or 40 μM Nec-1 markedly inhibited the NMDA-induced decrease in total retinal thickness and GCC thickness when compared with the NMDA + DMSO group. Meanwhile, immunofluorescence of retinal whole-mounts showed that the density of RGCs was markedly increased after administration of 80 μM GSK872 and 40 μM Nec-1 in the NMDA-injured eyes (Fig. 7b), indicating that GSK872 and Nec-1 could promote RGCs survival in excitotoxin-damaged mouse retinas.

**Fig. 8:**
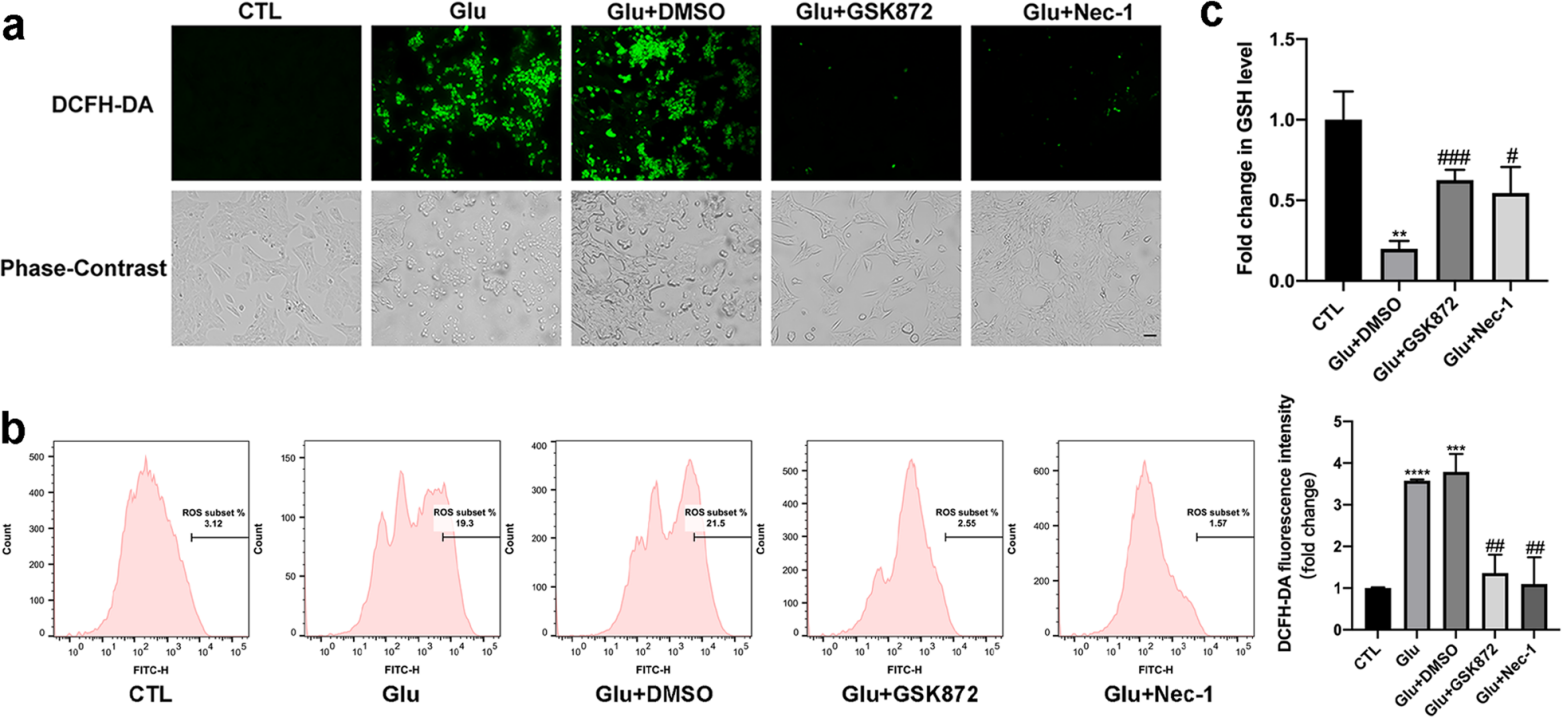
GSK872 and Nec-1 prevented glutamate-induced oxidative stress in R28 cells. **a** DCFH-DA probe was used to detect the intracellular ROS levels in R28 cells. The green fluorescence intensity indicated the level of intracellular ROS. Compared with the Glu + DMSO group, the fluorescence intensity was apparently decreased in the cells treated with GSK872 or Nec-1. Scale bar = 50 μm. **b** Flow cytometry was used to detect the DCFH-DA fluorescence density in R28 cells. **c** GSH levels in R28 cells were measured after administration of GSK872 or Nec-1. CTL: the control group; Glu: glutamate. The results were recorded as mean ± SD from at least three independent experiments. **p < 0.01, ***p < 0.001, ****p < 0.0001 versus control group; ^#^*p* < 0.05, ^##^*p* < 0.01, ^###^*p* < 0.001 versus Glu + DMSO group

We then measured intracellular ROS level changes with a DCFH-DA probe in R28 cells after the addition of GSK872 or Nec-1. In the fluorescence-stained images, significant inhibition of ROS production was observed in the GSK872- and Nec-1-treated groups compared with the Glu + DMSO group (Fig. 8a). Moreover, flow cytometry revealed that the increase in ROS triggered by glutamate was effectively suppressed by GSK872 and Nec-1 as well (Fig. 8b). Additionally, as a hallmark of the reduction system, cellular GSH levels were measured after the administration of GSK872 and Nec-1. The results showed that GSK872 and Nec-1 significantly increased GSH levels in R28 cells when compared with the Glu + DMSO group (Fig. 8c). Meanwhile, qRT-PCR revealed that distinct downregulation of TNF-α, IL-6, and IL-1β expression levels was observed in the cells incubated with Nec-1 compared with the Glu + DMSO group, while GSK872 suppressed the expression of IL-1β significantly but had a slight inhibitory effect on TNF-α and IL-6 (Additional file 2: Fig. S2). Taken together, the overproduction of ROS and the overexpression of proinflammatory cytokines triggered by glutamate could be prevented by GSK872 and Nec-1, as could the decrease in GSH, suggesting that the RIP1/RIP3/MLKL pathway contributes to glutamate-induced inflammation and oxidative stress.

**Fig. 9:**
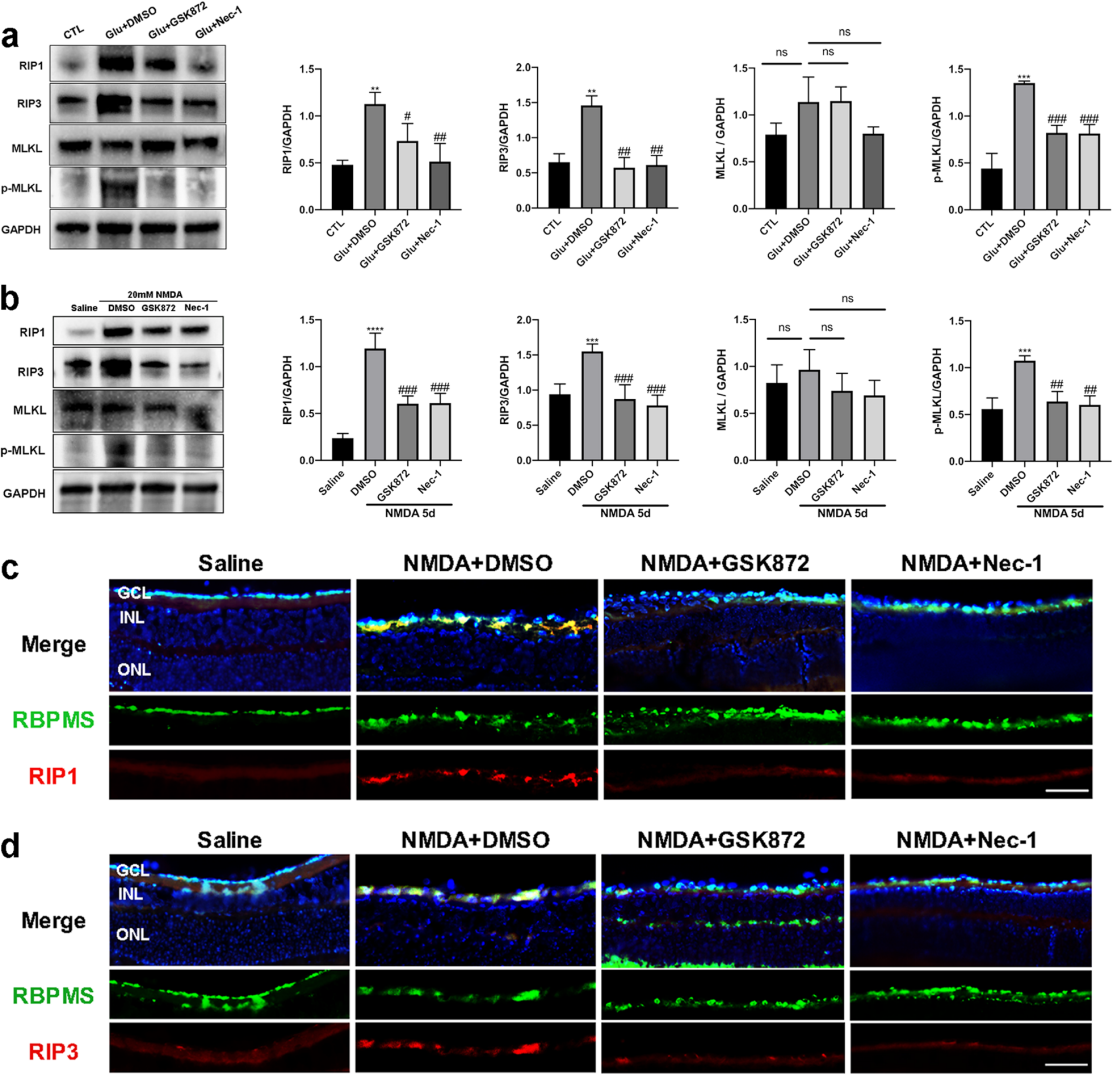
GSK872 and Nec-1 inhibited RIP1/RIP3/MLKL pathway activation induced by glutamate excitotoxicity. **a** Expression levels of RIP1, RIP3, MLKL and p-MLKL (phospho S345) in R28 cells after exposure to 10 mM glutamate for 24 h with or without the addition of GSK872 or Nec-1. **b** Intravitreal administration of GSK872 or Nec-1 downregulated protein levels of RIP1, RIP3 and p-MLKL (phospho S345) in NMDA-damaged eyes. **c** Representative immunofluorescence microphotographs of retinal sections stained with RBPMS (green), RIP1 (red), and DAPI (blue) at 5 days post GSK872 and Nec-1 injection. Scale bar = 50 μm. **d** Representative immunofluorescence microphotographs of retinal sections stained with RBPMS (green), RIP3 (red), and DAPI (blue) at 5 days post GSK872 and Nec-1 injection. Scale bar = 50 μm. GCL: ganglion cell layer; INL: inner nuclear layer; ONL: outer nuclear layer; CTL: the control group; Glu: glutamate. The results were recorded as mean ± SD from at least three independent experiments. **p < 0.01, ***p < 0.001, ****p < 0.0001 versus control or saline group; ^#^*p* < 0.05, ^##^*p* < 0.01, ^###^*p* < 0.001 versus Glu + DMSO group or NMDA + DMSO group. ns: not significant

Subsequently, we detected expression changes of RIP1, RIP3, MLKL and p-MLKL at the protein level after administration of GSK872 and Nec-1 in vitro and in vivo. In R28 cells, GSK872 and Nec-1 obviously decreased the expressions of RIP1, RIP3, and p-MLKL compared with the Glu + DMSO group, while there was no significant downregulation of MLKL (Fig. 9a). Simultaneously, the protein levels of RIP1, RIP3 and p-MLKL were markedly reduced by the intravitreal administration of GSK872 and Nec-1 in the NMDA-injured mouse retinas (Fig. 9b). Moreover, immunofluorescence analysis showed that apparent downregulation of RIP1 and RIP3 in RGCs was detected at 5 days after GSK872 and Nec-1 treatment, in contrast to the NMDA + DMSO group (Fig. 9c, d).

Consequently, combined with the results presented previously, we demonstrate that the RIP1/RIP3/MLKL pathway plays a vital role in regulating glutamate-induced retinal injury, oxidative stress, and necroptosis of RGCs, which can be blocked by the specific inhibitors GSK872 and Nec-1 in vivo and in vitro.

### Inhibition of RIP1/RIP3/MLKL pathway suppressed glutamate-triggered NLRP3 inflammasome activation

**Fig. 10:**
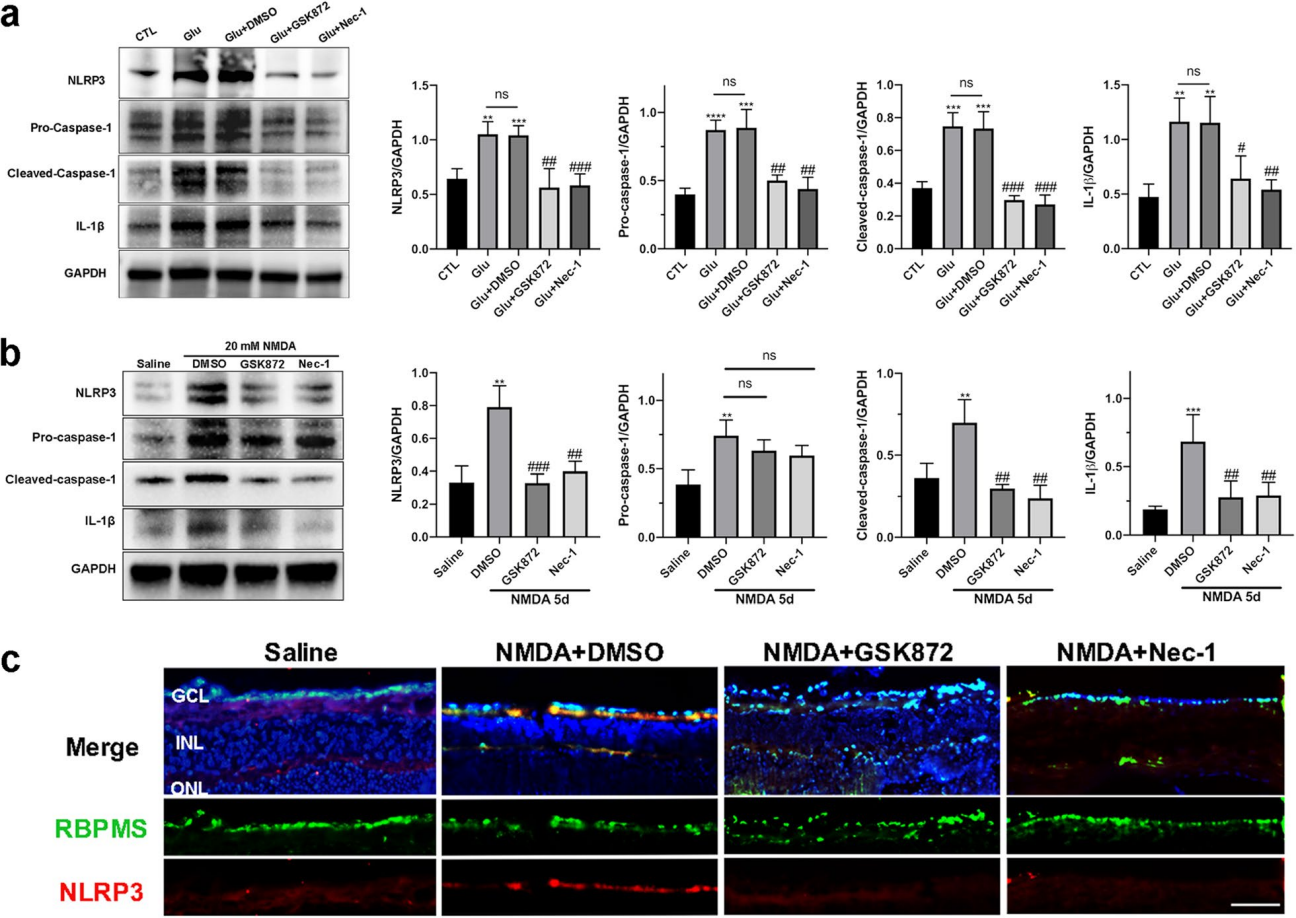
GSK872 and Nec-1 attenuated glutamate-induced NLRP3 inflammasome activation. **a** Expression levels of NLRP3 and NLRP3 downstream proteins in R28 cells after exposure to 10 mM glutamate for 24 h with or without the addition of GSK872 or Nec-1. **b** Intravitreal administration of GSK872 or Nec-1 significantly decreased protein levels of NLRP3 and NLRP3 downstream proteins in NMDA-damaged eyes. **c** Representative immunofluorescence microphotographs of retinal sections stained with RBPMS (green), NLRP3 (red), and DAPI (blue) at 5 days post GSK872 and Nec-1 injection. Scale bar = 50 μm. GCL: ganglion cell layer; INL: inner nuclear layer; ONL: outer nuclear layer; CTL: the control group; Glu: glutamate. The results were recorded as mean ± SD from at least three independent experiments. **p < 0.01, ***p < 0.001, ****p < 0.0001 versus control or saline group; ^#^*p* < 0.05, ^##^*p* < 0.01, ^###^*p* < 0.001 versus Glu + DMSO group or NMDA + DMSO group. ns: not significant

Previous studies have indicated that activated MLKL, the executor of necroptosis, plays a crucial role in NLRP3 inflammasome activation [47]. Thus, to investigate the underlying relationships between the RIP1/RIP3/MLKL pathway and NLRP3 inflammasome under glutamate treatment, we measured the expressions of key proteins in NLRP3 inflammasome activation, such as NLRP3, pro-caspase-1, cleaved-caspase-1, and IL-1β in R28 cells after GSK872 and Nec-1 administration. Robust elevation of NLRP3 protein level was observed in R28 cells after 24 h exposure to glutamate, and this upregulation could be significantly inhibited by GSK872 and Nec-1 (Fig. 10a). Similarly, the increased expressions of pro-caspase-1 and cleaved-caspase-1 induced by glutamate were markedly attenuated by GSK872 and Nec-1, as well, compared with the Glu + DMSO group (Fig. 10a). Moreover, GSK872 and Nec-1 could also prevent glutamate-induced upregulation of IL-1β at the mRNA and protein levels (Additional file 2: Fig. S2c, Fig. 10a). Meanwhile, our previous results showed that, as the downstream targets of NLRP3 inflammasome activation, the elevated mRNA levels of proinflammatory cytokines triggered by glutamate, such as TNF-α and IL-6, were obviously decreased upon NLRP3 inflammasome suppression (Additional file 2: Fig. S2).

Consistent with the in vitro results, intravitreal injection of GSK872 and Nec-1 remarkably inhibited NMDA-induced upregulation of NLRP3, cleaved-caspase-1, and IL-1β (Fig. 10b). Furthermore, we co-stained retinal cryosections with RBPMS and NLRP3 for immunofluorescence analysis, and the results showed that the fluorescence intensity of NLRP3 in RGCs was significantly decreased post GSK872 and Nec-1 treatment compared with the NMDA + DMSO group (Fig. 10c). Taken together, these results suggest that inhibition of the RIP1/RIP3/MLKL pathway by GSK872 and Nec-1 blocks glutamate-induced activation of NLRP3 inflammasome and subsequent proinflammatory cytokines release in vitro and in vivo. Thus, we could infer that the RIP1/RIP3/MLKL pathway contributes to glutamate-induced NLRP3 inflammasome activation, and it may be one of the upstream regulators of NLRP3 inflammasome activation in glaucoma.

## Discussion

Glaucoma is a group of optic neuropathies characterized by progressive RGCs degeneration and corresponding vision loss. Attributing to incomplete understanding of the pathogenesis, current treatments for glaucoma mainly focus on IOP reduction, which cannot reverse RGCs loss and optic nerve damage. Compelling evidence supports the idea that glutamate excitotoxicity plays an important role in RGCs death in glaucoma. Glutamate is the major excitatory neurotransmitter, mediating the transmission of visual signals by several retinal cell types, including bipolar cells, photoreceptors, and RGCs [4]. Under pathological conditions, excessive glutamate resulting from a disruption of the uptake and cycling system leads to the overstimulation of glutamate receptors (GluRs), inducing intracellular calcium overload, oxidative stress damage, neuroinflammation and RGCs death, which is called glutamate excitotoxicity [48]. GluRs in retinal neurons can be divided into metabotropic glutamate receptors (mGluRs) and ionotropic glutamate receptors (iGluRs), such as NMDA receptors [49, 50]. In many cases, the excessive stimulation of NMDA receptors contributes to the excitotoxic effect of glutamate [51]. Accordingly, NMDA receptors are regarded as potential therapeutic targets for glaucoma and other retinal degenerative diseases. However, memantine, an NMDA receptor antagonist, has failed in clinical trials for the treatment of glaucoma [52]. Thus, it is necessary to investigate the exact mechanisms of glutamate-induced RGCs death via NMDA receptors in glaucoma, which will provide a novel strategy to arrest glaucoma progression in addition to lowering IOP.

Necroptosis, a newly identified form of programmed cell death, has recently been regarded as an important contributor to neuronal death in many neurodegenerative diseases and retinal diseases, such as glaucoma and age-related macular degeneration (AMD). Inhibition of necroptosis has been suggested to attenuate ischemia-induced retinal damage, increased RGCs survival, and reduced hypoxia-induced RGCs death in a mouse model of retinal ischemic injury and an RGC cell model of hypoxic injury [53]. It is well documented that neurons exposed to glutamate undergo apoptosis and necrosis in vitro and in vivo [14, 54], but the role of necroptosis in glutamate-induced RGCs death remains elusive. In this study, we found that glutamate obviously inhibited cell viability, promoted LDH release, and increased cell death rates in R28 cells in a dose- and time-dependent manner. Importantly, we observed typical necroptotic morphological changes in R28 cells treated with glutamate and RGCs in NMDA-injured mouse eyes by using TEM. Similarly, in the NMDA-injected mouse retina, a significant decrease in retinal thickness and RGCs density was detected. In the HE staining analysis, we found that NMDA-induced injury was mainly restricted to the GCL and IPL, while the outer retina was less damaged. This may be attributed to the different expression of NMDARs in the retina. Previous studies have reported that NMDARs are robustly expressed in RGCs, as well as in some subsets of amacrine cells, whereas the expression of NMDARs in the outer retina, especially photoreceptors, has been poorly demonstrated [8]. Low expression of NMDARs in the outer retina prevents direct NMDA injury, but it remains to be investigated whether the cells of the outer retina are affected by toxic substances released by NMDA-damaged RGCs. Based on these results, we believe that necroptosis contributes to RGCs death caused by glutamate excitotoxicity in vitro and in vivo.

It has been well established that the RIP1/RIP3/MLKL pathway is the classic regulatory pathway of necroptosis under various circumstances [20]. After activation by stimuli such as ischemia/reperfusion, inflammation, and oxidative stress, RIP1 interacts with RIP3 to form a necrosome, which induces the activation and translocation of MLKL, leading to cell lysis. Therefore, to further investigate whether the classic necroptotic pathway is involved in glutamate-induced necroptosis in RGCs, we utilized the RIP3 specific inhibitor GSK872 and the RIP1 specific inhibitor Nec-1 in this study. Notably, we found that GSK872 and Nec-1 remarkably promoted cell survival in vitro and inhibited RGCs death as well as retinal thinning in vivo after excitotoxic damage. Furthermore, western blot analysis showed that the expression levels of RIP1, RIP3, and p-MLKL were significantly upregulated in both R28 cells and mouse retinas subjected to glutamate injury, but the increased protein levels could be obviously attenuated by GSK872 and Nec-1. In addition, consistent with the above results, immunofluorescence analysis of retinal sections revealed significant downregulation of RIP1 and RIP3 in RGCs post GSK872 and Nec-1 injection when compared with the NMDA + DMSO group. Taken together, our observations suggest that the RIP1/RIP3/MLKL pathway mediates necroptosis of RGCs caused by glutamate excitotoxicity both in vitro and in vivo. Additionally, GSK872 and Nec-1 can rescue RGCs from glutamate-induced necroptosis, demonstrating neuroprotective effects. However, in addition to the classical pathway, there are other mechanisms regulating necroptosis such as the apoptosis-inducing factor (AIF) dependent pathway [55–57]. It is worth further study to investigate whether other pathways and molecules contribute to glutamate-triggered necroptosis of RGCs.

In our study, we found that RIP1 was activated in the glutamate-induced excitotoxic glaucoma model in vivo and in vitro. However, the exact mechanism of RIP1 activation under NMDA overstimulation by glutamate remains unknown. It has been reported that in the human neuroblastoma cells, accumulation of cytosolic calcium induced by hemagglutinating virus of Japan-envelope (HVJ-E) can activate RIP1 and induce necroptosis through calcium-calmodulin kinase (CaMK) II phosphorylation of RIP1 [58], which indicates that accumulation of cytosolic calcium acts as an upstream activator of RIP1. So, it is possible that calcium influx induced by NMDA receptor activation may activate RIP1 through this way. However, other studies have demonstrated that calcium influx is triggered by RIP1 and RIP3 activation, and it is the downstream of necroptosis [59, 60]. Therefore, it remains to be further studied whether the NMDA receptor-dependent calcium influx can stimulate RIP1 in RGCs. In addition, it is well established that RIP1-dependent necroptotic pathway is triggered by the activation of death receptors (DRs) such as TNF receptor 1 (TNFR1) [61, 62]. Thus, it is also possible that calcium influx induced by NMDA receptor overstimulation can activate RIP1 through this TNFR1-mediated pathway, but the exact mechanisms are worth to be further investigated.

Growing evidence has demonstrated that ROS plays a vital role in glutamate-induced excitotoxicity. Previous studies have showed that in SH-SY5Y human neuroblastoma cells, glutamate could increase ROS and MDA levels, resulting in an oxidant–antioxidant imbalance that made cells vulnerable to oxidative stress [63]. In this study, we used fluorescence microscopy and flow cytometry to detect intracellular ROS levels in R28 cells with a DCFH-DA probe after glutamate exposure at different time points. The results showed that glutamate significantly promoted ROS production, which was consistent with previous research. Furthermore, to investigate the association between ROS and necroptosis, we measured ROS levels after GSK872 and Nec-1 administration. We found that overproduction of ROS induced by glutamate was significantly inhibited by GSK872 and Nec-1, suggesting that the RIP1/RIP3/MLKL pathway may regulate glutamate-triggered ROS elevation. However, conversely, intracellular ROS levels may also play a regulatory role in necroptotic activation. Lu et al. [64] discovered that in shikonin-treated glioma cells, inhibition of necroptosis could prevent intracellular ROS production, while mitigation of ROS with a cleaner of mitochondrial superoxide, MnTBAP, could attenuate shikonin-induced glioma cell necroptosis, as well as increase the expressions of RIP1 and RIP3. In contrast, increasing ROS levels with rotenone augmented necroptosis and upregulated expressions of RIP1 and RIP3. Given this, we speculated that there may be positive feedback between ROS and the necroptotic pathway. In addition to ROS, we found that glutamate-induced decrease in cellular GSH levels was significantly attenuated after administration of GSK872 and Nec-1, demonstrating that the RIP1/RIP3/MLKL pathway contributed to glutamate-induced oxidative stress in R28 cells.

NLRP3 inflammasome, composed of NLRP3, adaptor molecule apoptosis-associated speck-like protein containing a CARD (ASC) and caspase-1, is the most well characterized member of the inflammasome family and is reported to be constitutively expressed in the retinal pigment epithelium, ONH astrocytes, and retinal microglia and Müller cells [38, 65]. Previous studies have shown that increased mRNA and protein levels of IL-1β, a signal of NLRP3 inflammasome activation, were observed in the blood of glaucoma patients [66]. Furthermore, in a mouse model of acute glaucoma, activation of NLRP3 inflammasome was found to be required for RGCs death and inflammation [36], indicating that NLRP3 inflammasome plays a key role in RGCs loss and neuroinflammation in glaucoma. However, the underlying mechanisms and relationship between NLRP3 inflammasome activation and RGCs death remain elusive. The activation of NLRP3 inflammasome is very complicated, requiring two steps: the priming step and the activation step. In the priming step, expressions of NLRP3, pro-IL-1β, and pro-IL-18 are increased in response to stimuli, such as infection, injury, and ROS. The activation step results in oligomerization of ASC, NLRP3, and pro-caspase-1 to form the NLRP3 inflammasome, leading to the cleavage of pro-caspase-1, maturation of IL-1β, and subsequent cascades of inflammation [37]. An increasing number of studies have shown that necroptosis is closely associated with the NLRP3 inflammasome activation. Activated MLKL can translocate to the cell membrane and form pores, triggering potassium efflux, which is required for NLRP3 inflammasome activation [40]. The same study also suggested that MLKL-mediated NLRP3 and caspase-1 activation and the secretion of the proinflammatory cytokine IL-1β are major determinants of necroptotic-derived inflammatory signals [40]. Furthermore, Guo et al. [35] discovered that in lupus nephritis, necroptosis was accompanied by NLRP3 activation in vitro and in vivo. In the current study, to demonstrate the role of NLRP3 inflammasome in glutamate-induced RGCs death, as well as the relationship between the necroptotic pathway and NLRP3 inflammasome activation, we detected the levels of key proteins involved in NLRP3 inflammasome activation, including NLRP3, caspase-1, and IL-1β. Moreover, we measured the mRNA levels of TNF-α, IL-6, and IL-1β because proinflammatory cytokine release is the downstream of NLRP3 inflammasome activation. The results showed that the expressions of NLRP3, pro-caspase-1, cleaved-caspase-1, and IL-1β were remarkably increased in response to excitotoxic injury and exhibited a time-dependent action both in vitro and in vivo, which were significantly suppressed by GSK872 and Nec-1. Moreover, decreased fluorescence intensity of NLRP3 in the GCL was also detected following inhibition of the RIP1/RIP3/MLKL pathway by GSK872 and Nec-1 in the mouse retina. Meanwhile, glutamate-induced overexpression of proinflammatory cytokines was inhibited after intervention with GSK872 and Nec-1 in R28 cells. Consequently, our observations indicate that NLRP3 inflammasome activation is involved in RGCs death caused by glutamate excitotoxicity and is regulated by the RIP1/RIP3/MLKL necroptotic pathway. Meanwhile, inflammation following NLRP3 activation can also promote necroptosis in RGCs to a certain extent. Furthermore, it has been reported that NLRP3-dependent IL-1β release is a driver of necroptosis-induced inflammation. IL-1β released from necroptotic cells mediates the expression of a vast array of genes involved in secondary inflammation such as TNF-α and IL-6 via IL-1 receptor 1 (IR1), leading to inflammatory cascade in cells and RGCs death [37, 67]. Moreover, it has been shown that IL-1 plays an important role in animal models of blast mediated traumatic brain injury, and a specific antagonist of IL-1R1 anakinra resulted in protection of RGCs function and restoration of GCL thickness [68]. Based on these studies, we consider that NLRP3-dependent IL-1β plays a crucial role in inflammation and RGCs death in the glutamate-induced glaucoma model, while the exact pathway by which it triggers inflammation is worthy of further investigation. In addition to necroptosis, NLRP3 inflammasome activation plays a crucial role in pyroptosis [69], another type of programmed cell death mediated by the gasdermin-D (GSDMD) protein. GSDMD was discovered to be the substrate of caspase-1, and cleavage of GSDMD forms pores in cell membranes, driving pyroptosis [70]. Moreover, some morphological changes in pyroptosis are similar to those in necroptosis, such as cell swelling, pore formation, and membrane rupture [71]. Thus, whether pyrolysis is involved in glutamate-induced RGCs death requires further investigation.

In our study, we demonstrated the intrinsic necroptotic pathway and NLRP3 inflammasome axis in R28 cells and RGCs in the glutamate-induced excitotoxic glaucoma model. However, in the process of glaucoma, it is believed that RGCs death results from a system of complex interactions of many different cells such as microglia and astrocyte [67, 72]. So, it is possible that cell–cell contact such as RGC–microglia contact may be involved in this the association between necroptosis and NLRP3 inflammasome. Microglia, the immunocompetent cells that perform immune surveillance and mediate inflammatory responses to infection, disease, or injury in the CNS and retina [73], has been reported to play a vital role in RGCs loss and neuroinflammation in glaucoma [74]. Under pathological conditions, overactivation of microglia induces production of proinflammatory cytokines such as TNF-α, IL-6, and IL-1β [75]. In a mouse model of inherited glaucoma, DBA/2J mice, the extent of neurodegeneration correlated with early microglial alterations in vivo, which could be inhibited by minocycline, inhibitor of microglial activation [74, 76]. In addition, studies have shown that microglia activation mediated RGCs loss in the NMDA-induced excitotoxic mouse model via production of TNFα and activation of TNF receptor 1 (TNFR1), which could be inhibited by pharmacological inhibition and deletion of microglia and genetic ablation of microglia [77]. It is well established that necroptosis can be triggered by the activation of death receptors such as TNFR1 [61, 62]. Therefore, it is logical to speculate that necroptosis of RGCs was correlated to microglia activation. In the NMDA-induced excitotoxic glaucoma model, microglia were activated and the release of TNFα from activated microglia may spread around RGCs and activate the TNFR1 of RGCs, leading to RIP1/RIP3/MLKL pathway activation and following RGCs necroptosis. In our study, we discussed that necroptotic pathway acted as an intrinsic activator of NLPR3 inflammasome in RGCs. However, NLPR3 inflammasome can also be activated by cell-extrinsic signals, including innate immune signaling or cytokine receptors, such as the TNFR [78]. Thus, the stimulation of TNFR1 induced by microglia activation may also play a role in the glutamate-induced NLRP3 inflammasome activation in RGCs, but the exact mechanisms remain to be further studied.

In addition to the effect microglia have on RGCs, RGCs also play an important role in microglia activation. Studies have reported that ATP released from NMDA-stimulated RGCs can activate microglia via P2X7 receptors, leading to TNFα releasing [79, 80]. Therefore, in the NMDA-induced excitotoxic model, microglia may be activated by stressed RGCs because microglia do not express functional NMDA receptors and cannot be triggered by NMDA directly [77]. Then, the activated microglia can induce RGCs necroptosis, forming a complex cross-talk. Besides microglia, other cell types including astroglia, Müller cells and infiltrating leukocytes participate in RGCs death and have interactions with RGCs [75], and they may also play a role in the necroptosis/NLPR3 axis of RGCs, which needs to be further investigated. It is of great significance to understand the correlations between RGCs and other retinal cells, helping to develop novel RGCs protection strategies.

## Conclusions

**Fig. 11:**
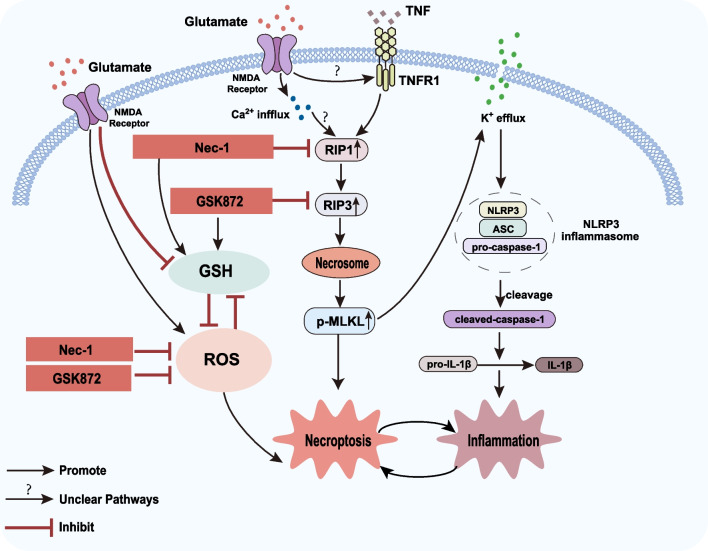
Potential molecular mechanisms underlying glutamate-induced RGCs necroptosis and NLRP3 inflammasome activation. GSK872 and Nec-1 prevent RGCs from necroptosis and suppressed NLRP3 inflammasome activation and ROS overproduction by inhibiting the RIP1/RIP3/MLKL pathway in a glutamate-induced excitotoxic glaucoma model

Our results indicate that necroptosis may play a vital role in RGCs death in glaucoma. GSK872 and Nec-1 can protect RGCs from necroptosis, inhibit NLRP3 inflammasome activation and oxidative stress by suppressing the RIP1/RIP3/MLKL pathway in a glutamate-induced excitotoxic glaucomatous model (Fig. 11). This study provides novel insights into the neuroprotective treatment for glaucoma.

## Materials and methods

### Cell culture

The R28 retinal precursor cell line, an immortalized adherent retinal precursor cell line derived from infantile Sprague–Dawley rat retinas, is used for in vitro studies of neuroprotection, cytotoxicity, and physiological function of RGCs [10–12, 81]. The R28 cells were cordially provided by the Department of Anatomy and Neurobiology of Central South University (Changsha, China). The R28 cells were cultured in low-glucose DMEM (Gibco, Waltham, Massachusetts, USA) with 10% FBS (Cell-Box, China) and 1% penicillin/streptomycin (Gibco, Waltham, Massachusetts, USA). The cells were maintained in a humidified incubator at 37 ℃ and 5% CO_2_.

### Animals

This study used 140 7-week-old (20–25 g) C57BL/6J mice. The animals were maintained under standard laboratory conditions of a 12-h cycle of light and dark at a temperature of 21 ± 1 ℃. Food and water were available ad libitum. All animals were treated according to the Association for Research in Vision and Ophthalmology Resolution on the Use of Animals in Research. The mice were randomly divided into seven groups of 20 mice each: saline group, NMDA 1 d group, NMDA 3 d group, NMDA 5 d group, NMDA + DMSO group, NMDA + GSK872 group, NMDA + Nec-1 group. The retinal excitotoxic damage model was achieved by intravitreal injection of 1 μL of 20 mM NMDA in saline (APExBIO Technology, Houston, TX, USA). Mice were anesthetized with an intraperitoneal injection of 1% pentobarbital sodium (10 mL/kg), followed by oxybuprocaine hydrochloride eye drops for topical anesthesia. Pupils were dilated with tropicamide eye drops. Subsequently, 1 μL NMDA was injected into the vitreous cavity of both eyes using a 33-gauge needle connected to a 10-µL syringe (Hamilton Company, USA). The control group received intravitreal injection with the same volume of saline. Antibiotic ophthalmic ointment was applied to the eyes post-injection. The animals were killed at 1, 3, and 5 days after intravitreal injection, and the eyes were enucleated and processed for further analysis.

### Drug administration

For the in vitro analysis, R28 cells were seeded in 6-well plates at a density of 3 × 10^5^ cells/well and incubated overnight before treatment with different concentrations of glutamate (Abcam, Cambridge, UK) and for different durations. GSK872 (Selleck, Houston, TX, USA) and Nec-1 (Sigma-Aldrich, St. Louis, MO, USA) were dissolved in DMSO and diluted in culture medium. The cells were divided into five groups according to different treatments: Control group (without treatment), Glutamate-treated, Glutamate + DMSO treated, Glutamate + GSK872 treated, and Glutamate + Nec-1 treated groups. Then the cells were incubated for 24 h before being analyzed.

For the in vivo analysis, mice received an intravitreal injection of 1 μL solution containing NMDA and GSK872 (80 μM in saline) or NMDA and Nec-1 (40 μM in saline). The group treated with the same volume of solution containing NMDA and DMSO was used as control. All treatments were given intravitreally in both eyes. Five days after injection, eyes were enucleated and processed for further analysis.

### Observation of cell morphology

The R28 cells were seeded in 6-well plates at a density of 3 × 10^5^ cells/well and incubated overnight. Then the cells were exposed to 10 mM glutamate for 6 h, 12 h, and 24 h. At each time point, cells were observed and photographed using phase-contrast microscopy (Nikon, Tokyo, Japan).

### Cell viability assay

Cell viability was measured using a Cell Counting Kit-8 (CCK-8, NCM Biotech, China) according to the manufacturer’s instructions. The R28 cells were seeded at a density of 1 × 10^4^ cells/well in 96-well plates and incubated overnight. Then the cells were treated with different concentrations of glutamate, GSK872, and Nec-1 for 24 h. Subsequently, 10 μL CCK-8 solution was added to each well and incubated for 2 h. The absorbance was measured at 450 nm using a spectrophotometer.

### Lactate dehydrogenase release assay

An LDH release assay kit (Beyotime, Shanghai, China) was used to measure LDH released from necrotic cells after different treatments. In brief, cell cultures were collected from 96-well plates after being centrifuged at 400*g* for 5 min using a porous plate centrifuge and then the cell-free culture supernatants were incubated with the working reagent mixture in the dark for 30 min at room temperature according to the manufacturer’s instructions. Subsequently, the absorbance was measured with a spectrophotometer at a wavelength of 490 nm. The LDH release rate of R28 cells was calculated as the percentage of the absorbance value: LDH release rate% = (sample cells − control cells)/(LDH releasing reagent treated cells − control cells) × 100.

### Hoechst 33342/PI dual staining assay

Hoechst 33342/PI dual staining (Beyotime, Shanghai, China) was used to measure the cell death rate of the R28 cells. After being treated with the target compounds, the R28 cells were incubated with cell staining buffer, Hoechst staining solution, and PI staining solution at 4 ℃ in the dark for 30 min according to the manufacturer’s instructions. After being washed with PBS, the cells were observed and photographed with a fluorescence microscope (Nikon, Tokyo, Japan). The photos were analyzed using ImageJ software. The percentage of cell death was calculated using the formula: cell death rate% = PI-positive cells/Hoechst-positive cells × 100. Five visual fields were selected in each sample.

### Annexin V-FITC/PI double staining

Annexin V-FITC detection kit (Beyotime, Shanghai, China) was used to detect apoptosis and necrosis in R28 cells after glutamate treatment. After incubation with glutamate for different time points, R28 cells were collected using EDTA-free trypsin and then resuspended with PBS. After that, cells were stained with 5 μL of Annexin V-FITC and 10 μL of PI for 15 min at room temperature in the dark according to the manufacturer’s instructions. Next, the stained cells were analyzed by flow cytometry. The percentage of Annexin V^+^ cells indicated cell death rate, which was consisted of Annexin V^+^/PI^−^ (early apoptosis) cells and Annexin V^+^/PI^+^ (necrosis and late apoptosis) cells while both Annexin V and PI negative cells were considered as living cells. For each sample, 3 × 10^4^ cells were collected.

### Transmission electron microscopy (TEM)

After being treated with the target compounds, R28 cells and retinas were harvested and then fixed in ice-cold 2.5% glutaraldehyde at 4 ℃ in the dark for 24 h. After being washed with PBS 3 times for 10 min, cells and retinas were fixed with 1% osmium tetroxide for 3 h, and then rinsed with PBS 3 times for 10 min. Next, the samples were gradient dehydrated with ethanol and acetone and embedded in epoxy resin followed by being cut into 50–60 nm sections. Then, the sections were observed under a HT7700 transmission electron microscope (HITACHI, Tokyo, Japan) after being stained with 3% uranyl acetate combined with lead citrate.

### ROS production detection

The intracellular ROS level was measured using an ROS assay kit (Biosharp, China). In brief, after being treated with different compounds, the R28 cells were stained with 10 μM DCFH-DA at 37 ℃ in the dark for 30 min according to the manufacturer’s instructions. Then, the cells were observed and photographed under a fluorescence microscope (Nikon, Tokyo, Japan) after being washed three times with fresh medium. Moreover, the fluorescence intensity of DCFH-DA was measured by flow cytometry.

### qRT-PCR

Total RNA was extracted from the R28 cells using TRIzol reagent (Invitrogen, Waltham, MA, USA), and cDNA was synthesized with a Hifair III 1st Strand cDNA Synthesis Kit (Yeasen Biotechnology, Shanghai, China). Quantitative real-time polymerase chain reaction (RT-PCR) was performed using a Hieff qPCR SYBR Green Master Mix (Low Rox, Yeasen Biotechnology, Shanghai, China) with a sequence detection system (Prism 7500, Applied Biosystems, Waltham, MA, USA) according to the manufacturer’s instructions. The specific primers were designed by Sangon Biotech (Shanghai, China), including TNF-α (forward 5′-ACCATGAGCACGGAAAGCAT-3′ and reverse 5′-AACTGATGAGAGGGAGCCCA-3′), IL-6 (forward 5′-TCTGGTCTTCTGGAGTTCCGT-3′ and reverse 5′-CTTGGTCCTTAGCCACTCCT-3′), IL-1β (forward 5′-TACCTATGTCTTGCCCGTGG-3′ and reverse 5′-TAGCAGGTCGTCATCATCCC-3′), and GAPDH (forward 5′-AGTGCCAGCCTCGTCTCATA-3′ and reverse 5′-TGAACTTGCCGTGGGTAGAG-3′). Relative mRNA levels were normalized to those of GAPDH and were calculated using the 2^−ΔΔCT^ method. Each sample was measured in triplicate wells, and the experiments were repeated three times.

### Western blot analysis

After being treated with the target compounds, proteins from the R28 cells and retinas were harvested using a RIPA lysis buffer (Beyotime, Shanghai, China) with Protease Inhibitor Cocktail (APExBIO Technology, Houston, TX, USA) and Phosphatase Inhibitor Cocktail (APExBIO Technology, Houston, TX, USA), and then the retinas were homogenized at 4 ℃ for 15 min. Next, the lysates were centrifuged at 12,000*g* and 4 ℃ for 20 min. Then, the supernatant was transferred into a fresh tube and subjected to sonication, clarified by centrifugation (12,000*g*, 4 ℃ for 10 min), and the supernatant was collected. The protein concentration was measured by bicinchoninic acid assay (Beyotime, Shanghai, China). After being mixed with 5 × loading buffer and boiled at 95 ℃ for 10 min, the protein was subjected to electrophoresis on an SDS/PAGE gel and transferred to PVDF membranes. After blocking with 5% skim milk for 90 min, the membranes were incubated overnight at 4 ℃ with the following primary antibodies: anti-RIP1 (#3493, 1:1000, Cell Signaling Technology, Danvers, MA, USA), anti-RIP3 (#15,828, 1:1000, Cell Signaling Technology, Danvers, MA, USA), anti-MLKL (ab243142, 1:2000, Abcam, Cambridge, UK), anti-MLKL(phospho S345) (ab196436, 1:1000, Abcam, Cambridge, UK), anti-NLRP3 (ab263899, 1:1000, Abcam, Cambridge, UK), anti-caspase-1 (22915-1-AP, 1:2000, Proteintech, Rosemont, IL, USA), anti-IL-1β (ab254360, 1:1000, Abcam, Cambridge, UK), anti-GAPDH (60004-1-Ig, 1:20000, Proteintech, Rosemont, IL, USA). Then, the membranes were washed three times with TBST for 10 min and incubated with the respective peroxidase-conjugated secondary antibodies (Zen Bio, Chengdu, China) for 2 h at room temperature. After three 10-min washes, the proteins were visualized using an enhanced chemiluminescence (ECL) method (NCM Biotech, China) with a chemiluminescence imager (Bio-Rad, California, USA) according to the manufacturer’s instructions. The integrated optical density values of specific proteins were quantified using ImageJ software. GAPDH was used as the internal control.

### Measurement of glutathione (GSH)

GSH was detected using a GSH and GSSG Assay Kit (Beyotime, Shanghai, China). In brief, cells were collected after being washed with PBS and then mixed with the protein removal reagent (M) solution. Then, the samples were freeze–thaw twice using liquid nitrogen and 37° water bath followed by ice bath for 5 min. Next, several working solutions of the assay were prepared according to the manufacturer’s instructions. After incubation with the working solutions for the indicated times, GSH levels were measured by detecting the absorbance value of all samples at 412 nm using a spectrophotometer.

### Hematoxylin and eosin (HE) staining

After the mice were killed, their eyes were enucleated at the designated time points, fixed with 4% paraformaldehyde (PFA), and embedded in paraffin. Four paraffin-embedded retinal tissue sections of each eye were cut in a vertical meridian through the optic disk at 4 µm thickness and stained with HE. Micrographs of the stained retinal sections were captured using a light microscope (Leica, Wetzlar, Germany). Total retinal thickness (the internal to the outer limiting membrane) and GCC thickness (from the INL to the nerve fiber layer) were measured within the area of 1 mm distance to the optic nerve. Each measurement was performed four times and averaged.

### Immunofluorescence staining of retinal whole-mounts

At different time points post intravitreal injection, the mice were killed and perfused with saline and 4% PFA. Eyes were then enucleated and fixed in 4% PFA for 2 h at room temperature. Subsequently, retinas were isolated from the eyecups, and each retina was divided into four quadrants. Next, retinas were incubated with blocking buffer containing 5% bovine serum albumin (BSA) and 0.5% Triton-X 100 in PBS (PBS-T) for 90 min at room temperature. Then, retinas were incubated overnight at 4 ℃ with guinea pig anti-RBPMS (#1832-RBPMS,1:100, PhosphoSolutions, Denver, CO, USA) in blocking buffer. After being fixed in 4% PFA for 10 min and washed three times in PBS-T, the retinas were incubated with the secondary antibody (goat anti-guinea pig Alexa Fluor 488, ab150185, 1:1000, Abcam, Cambridge, UK) in blocking solution for 90 min at room temperature. Finally, retinas were washed in PBS three times and mounted with the mounting medium containing DAPI. Then, retinal whole-mounts were examined with a fluorescence microscope (Leica, Wetzler, Germany). RBPMS-positive RGCs were quantified by two observers using ImageJ software in three areas (center, middle, periphery) in each of the four quadrants of the retina.

### Immunofluorescence staining of retinal sections

Retinal cryosections were prepared for immunofluorescence according to standard techniques. Briefly, the mice were killed, and eyes were enucleated and fixed in 4% PFA overnight at 4 ℃. Then, the eyes were incubated in 10%, 20%, and 30% sucrose in PBS and frozen in optimum cutting temperature (OCT) compound (Sakura Finetek, Torrance, CA, USA). Ocular tissue sections were cut through the optic disk of each eye using a cryostat at a thickness of 15 µm and dried on glass slides. Subsequently, cryosections were fixed with 4% PFA for 10 min at room temperature and then blocked with blocking solution (5% BSA in PBS-T) for 30 min. Next, the sections were incubated with the following primary antibodies diluted in the blocking solution overnight at 4 ℃: guinea pig anti-RBPMS (#1832-RBPMS,1:100, PhosphoSolutions, Denver, CO, USA), rabbit anti-RIP1 (#3493, 1:100, Cell Signaling Technology, Danvers, MA, USA), rabbit anti-RIP3 (#95702, 1:100, Cell Signaling Technology, Danvers, MA, USA), and rabbit anti-NLRP3 (#DF7438, 1:100, Affinity Biosciences, USA). Following the primary incubation, sections were stained with the secondary antibodies (goat anti-guinea pig Alexa Fluor 488, ab150185,1:1000, Abcam, Cambridge, UK and goat anti-rabbit Alexa Fluor 647, ab150079, 1:1000, Abcam, Cambridge, UK) for 90 min at room temperature and then mounted with the mounting medium containing DAPI. Images were obtained with a fluorescence microscope (Leica, Wetzler, Germany).

### Statistical analysis

All data are presented as mean ± standard deviation (SD) from at least three independent experiments. Statistical analysis was performed using the GraphPad Prism software (version 8.0). Multiple data were analyzed using one-way analysis of variance (ANOVA), followed by Tukey’s multiple comparison test. Student’s t-test was also performed to analyze the differences between the two groups. Differences were considered statistically significant at *p* < 0.05.

## Supplementary Information

**Additional file 1: Figure S1.** Overproduction of proinflammatory cytokines induced by glutamate in R28 cells. qRT-PCR was used to detect the expression levels of TNF-α, IL-6, and IL-1β in R28 cells subjected to glutamate at 6 h,12 h, and 24 h. CTL: the control group; Glu: glutamate. The results were recorded as mean ± SD from at least three independent experiments. *p < 0.05, **p < 0.01, ***p < 0.001, ****p < 0.0001 versus control group.

**Additional file 2: Figure S2**. GSK872 and Nec-1 inhibited glutamate-induced upregulation of proinflammatory cytokines. qRT-PCR was used to detect the expression levels of TNF-α, IL-6, and IL-1β in R28 cells after GSK872 and Nec-1 administration. CTL: the control group; Glu: glutamate. The results were recorded as mean ± SD from at least three independent experiments. **p < 0.01, ***p < 0.001 versus control group; ^#^*p* < 0.05, ^##^*p* < 0.01 versus Glu + DMSO group. ns: not significant.

## Data Availability

The datasets used and/or analyzed during the current study are available from the corresponding author on reasonable request.

## Abbreviations

RGCs: Retinal ganglion cells
NMDA: *N*-Methyl-d-aspartic acid
Glu: Glutamate
CTL: Control group
NLRP3: Nucleotide-binding leucine-rich repeat-containing receptor family, pyrin domain containing 3
RIP1: Receptor interacting protein kinase 1
RIP3: Receptor interacting protein kinase 3
MLKL: Mixed lineage kinase-like domain protein
IOP: Intraocular pressure
Nec-1: Necrostatin-1
ROS: Reactive oxygen species
GSH: Glutathione
IL-1β: Interleukin-1β
IL-6: Interleukin-6
GCL: Ganglion cell layer
IPL: Inner plexiform layer
INL: Inner nuclear layer
OPL: Outer plexiform layer
ONL: Outer nuclear layer
GCC: Ganglion cell complex
TEM: Transmission electron microscopy
