# Supplementary figures and images for: GSK872 and necrostatin-1 protect retinal ganglion cells against necroptosis through inhibition of RIP1/RIP3/MLKL pathway in glutamate-induced retinal excitotoxic model of glaucoma
Source: J Neuroinflammation. 2022 Oct 26;19:262. doi: 10.1186/s12974-022-02626-4 (PMC9608931; doi:10.1186/s12974-022-02626-4)

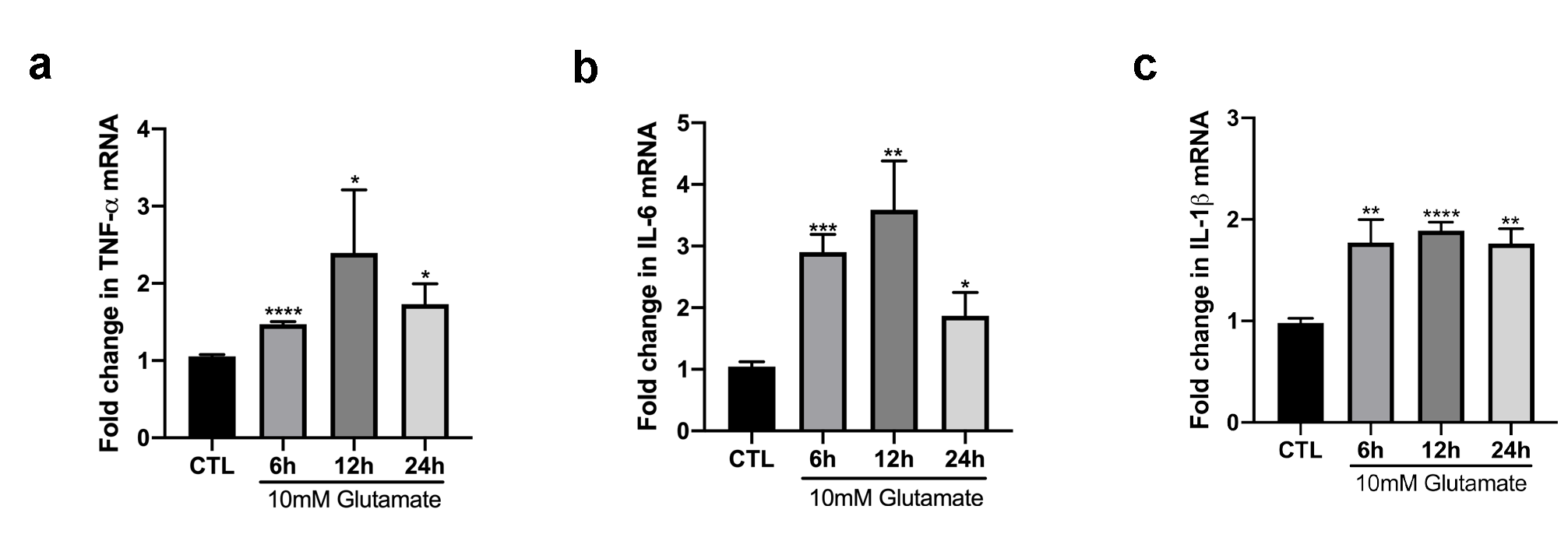

Supplement: Supplementary file 1 — Additional file 1: Figure S1. Overproduction of proinflammatory cytokines induced by glutamate in R28 cells. qRT-PCR was used to detect the expression levels of TNF-α, IL-6, and IL-1β in R28 cells subjected to glutamate at 6 h,12 h, and 24 h. CTL: the control group; Glu: glutamate. The results were recorded as mean ± SD from at least three independent experiments. *p < 0.05, **p < 0.01, ***p < 0.001, ****p < 0.0001 versus control group. [file 12974_2022_2626_MOESM1_ESM.tif]

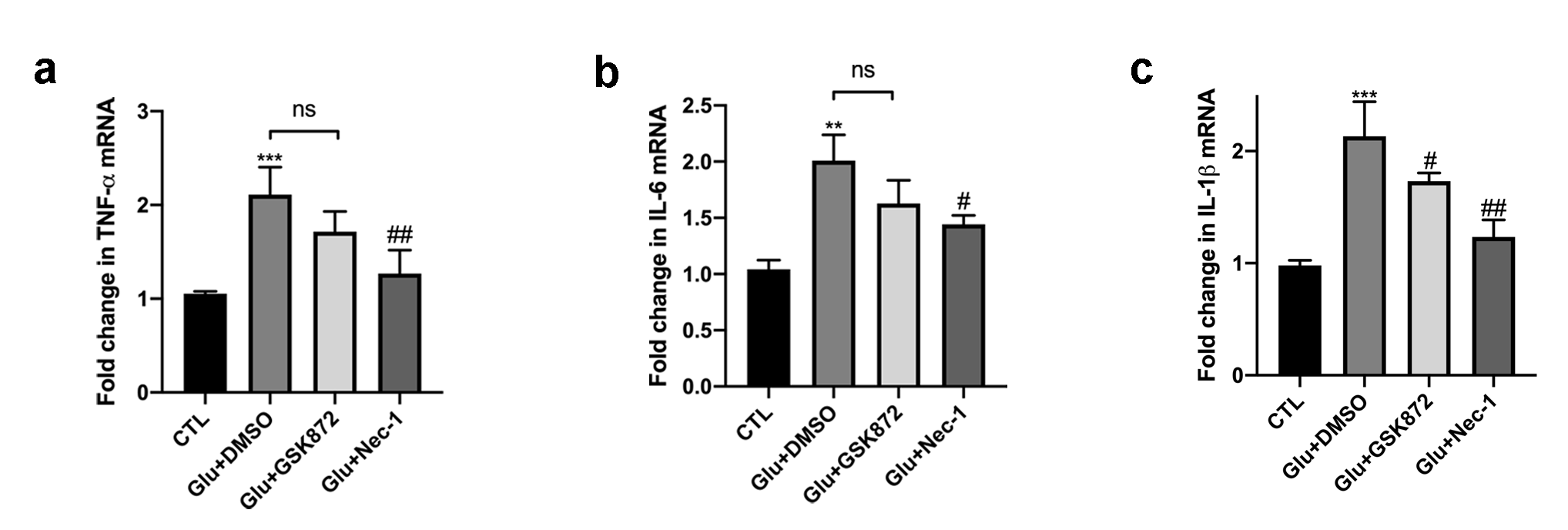

Supplement: Supplementary file 2 — Additional file 2: Figure S2. GSK872 and Nec-1 inhibited glutamate-induced upregulation of proinflammatory cytokines. qRT-PCR was used to detect the expression levels of TNF-α, IL-6, and IL-1β in R28 cells after GSK872 and Nec-1 administration. CTL: the control group; Glu: glutamate. The results were recorded as mean ± SD from at least three independent experiments. **p < 0.01, ***p < 0.001 versus control group; #p < 0.05, ##p < 0.01 versus Glu + DMSO group. ns: not significant. [file 12974_2022_2626_MOESM2_ESM.tif]
